# Chemical Composition, Biomolecular Analysis, and Nuclear Magnetic Resonance Spectroscopic Fingerprinting of *Posidonia oceanica* and *Ascophyllum nodosum* Extracts

**DOI:** 10.3390/metabo13020170

**Published:** 2023-01-24

**Authors:** Angelica Bruno, Aldrik H. Velders, Alessandro Biasone, Mario Li Vigni, Donato Mondelli, Teodoro Miano

**Affiliations:** 1 Department of Soil, Plant and Food Sciences, Faculty of Agricultural Science, University of Bari Aldo Moro, 70126 Bari, Italy; 2 Laboratory of BioNanoTechnology, Wageningen University and Research, 6708 WG Wageningen, The Netherlands; 3 Global R&D Department, Valagro SpA, 66041 Atessa, Italy; 4 CIHEAM, Mediterranean Agronomic Institute of Bari, Via Ceglie 9, 70010 Valenzano, Italy

**Keywords:** *Posidonia oceanica*, *Ascophyllum nodosum*, extraction methods, chemical composition, plant metabolic profile, NMR spectroscopy

## Abstract

A detailed analysis of the elemental and molecular composition of *Posidonia oceanica* (PO) and *Ascophyllum nodosum* (AN) is presented. In particular, an in-depth study of the molecular identification via NMR spectroscopy of aqueous and organic extracts of PO and AN was carried out, exploiting 2D COSY and pseudo-2D DOSY data to aid in the assignment of peaks in complex 1D proton NMR spectra. Many metabolites were identified, such as carbohydrates, amino acids, organic acids, fatty acids, and polyphenols, with NMR complementing the characterization of the two species by standard elemental analysis, HPLC analysis, and colorimetric testing. For PO, different parts of the live plant (roots, rhizomes, and leaves) were analysed, as well as the residues of the dead plant which typically deposit along the coasts. The combination of the various studies made it possible to recognize bioactive compounds naturally present in the two plant species and, in particular, in the PO residues, opening the door for their possible recycling and use in, for example, fertilizer. Furthermore, NMR is proven to be a powerful tool for the metabolomic study of plant species as it allows for the direct identification of specific biomarkers as well as providing a molecular fingerprint of the plant variety.

## 1. Introduction

*Posidonia oceanica* (L.) Delile is a widespread endemic plant in the Mediterranean Sea and is found, in particular, in the coastal waters of southern Italy. From an ecological point of view, this marine plant constitutes an environment of extraordinary importance for the Mediterranean region, carrying out multiple functions: it is involved in the oxygenation of marine waters and in the protection of sandy coasts from erosion, and it represents an ideal habitat for many organisms, contributing to the formation of coastal dunes and providing an important trophic resource for many fish [1,2,3]. Like other higher plants, PO cyclically loses leaves that partly remain in the sea, re-entering the food chain and the sedimentation cycles; in part, leaves are also deposited along the shoreline, forming structural deposits called *banquettes*. Besides the fundamental ecological role of PO in the marine coastal ecosystem, the decomposition of its organic residues causes, in addition to the unpleasant aesthetic appearance of the coastline, the development of bad smells and the spread of flies, thus compromising, e.g., tourist activity and specific environmental quality. The removal of these plant residues from the coastline commonly implies the use of heavy machinery and disposal in controlled landfills with concomitant economic costs and environmental problems. Alternative uses of the PO deposits currently under investigation comprise their application as a mulch and soil conditioner, in the production of compost or biogas [4], in the production of cellulose or material for food packaging [5], for use as insulating material [6], and for pharmacological products in the prevention or treatment of various diseases [7,8,9]. In this perspective, here, this marine species and its related biomass waste were evaluated for its potential use in, e.g., agriculture. The elemental and molecular analysis of various parts of the PO were compared with *Ascophyllum nodosum* (AN), one of the most used brown algal species for the beneficial properties of its bioactive components. The AN extract was shown to possess numerous stimulating properties for plants, i.e., in the stimulation of germination, growth enhancement, high resistance to biotic and abiotic stresses, and an improved post-harvest shelf life [10,11,12,13]. A comparison was then made between the two species to evaluate whether bioactive compounds can also be obtained from PO to be used, e.g., in agriculture, as is already performed for AN.

Besides the classical chemical characterization pursued using well-established analytical techniques, nuclear magnetic resonance (NMR) spectroscopy was used for the first time to build a molecular fingerprint of the two species for identification and comparison. The rising capacity of high-field NMR methods for molecular identification in complex mixtures of different compounds without physically separating them has found many new applications in metabolomics and food science [14,15]. In fact, unlike most molecular analysis techniques, NMR spectroscopy does not need time-consuming chemical pre-treatments of the sample (such as fractionations, purification, hydrolyses, derivatizations) and, in most cases, can produce qualitative and quantitative information on the initial extracts. Moreover, NMR has many other advantages: (i) the technique is universal and impartial as it is not limited to a single class of metabolites; (ii) the sample is not destroyed during NMR measurement and data acquisition are relatively rapid; (iii) the identification of known compounds is relatively straightforward and unknowns or unexpected compounds can often be identified as well; (iv) signals are proportional to their molar concentrations, making direct comparisons of the concentrations of compounds, and quantitative data are obtained relative to a single standard without the need for calibration curves; and (v) NMR spectra can function as molecular fingerprints.

Standard one-dimensional ^1^H-NMR spectra of the aqueous and organic extracts of the two species studied here can be rather complex because they represent mixtures of different compounds, which by themselves typically contain multiple unique hydrogen atoms giving a corresponding number of peaks. For most sample mixtures obtained in our study, the resolution of mono-dimensional NMR spectra appeared insufficient for the unambiguous assignment of the peaks. Therefore, complementary NMR techniques were used in association, such as ^1^H-^1^H COrrelated SpectroscopY (COSY) and Diffusion-Ordered SpectroscopY (DOSY). COSY provides 2D NMR spectra that consist of off-diagonal correlation peaks or “cross peaks” which provide molecular information, as they indicate protons belonging to the same molecule via scalar through-bond couplings. Similarly, Diffusion-Ordered SpectroscopY (DOSY) experiments allow to discriminate the signals from different compounds in a mixture based on their respective specific diffusion coefficients, depending on the size, shape, and chemical structure of the molecules [16,17]. In this way, by combining the information obtained from these different NMR experiments, it is possible to resolve, at least in part, very complex spectra and identify a wide range of molecules present in various plant extracts.

For the analyses of the two species (PO and AN), four different extraction methods were used (acid, alkaline, hydroalcoholic, and alcoholic extraction) to selectively isolate and characterize specific categories of chemicals, using appropriate solvent mixtures, pH, and temperature. Furthermore, regarding the PO, a comparison was made between the live plant (divided into different parts such as the roots, including rhizomes, and leaves) and its coastal residues. The results of the NMR analysis were complemented with the results of the conventional techniques for carbohydrate, amino acid, and polyphenolic content. The aim of this study was primarily to characterize, more completely and comprehensively, the marine plant *Posidonia oceanica* in comparison with the seaweed *Ascophyllum nodosum*, to evaluate their content in bioactive compounds and hence potential application in agriculture to improve the growth and quality of vegetable crops. To this end, our research exploited NMR spectroscopy as a technique for identifying and comparing the molecular profiles of the two species and more specifically even different fractions of the plant. In particular, this research supports the possible reuse and valorisation of PO waste biomass.

## 2. Materials and Methods

### 2.1. Materials

All reagents were of analytical grade unless otherwise stated. Calibration curves for ICP analysis were obtained using the ICP standard solution of B, Ca, Cu, Fe, K, Mg, Mo, Mn, Na, P, and Zn purchased from Merck (Darmstadt, Germany). The HCl, HNO_3_, and H_2_O_2_ were purchased from Sigma-Aldrich (Germany). All solutions were prepared using high purity water from a Milli-Q Element system (Millipore, Molsheim, France). HPLC grade methanol and acetonitrile were obtained from Merck (Italy). The amino acid standards, 2-aminoadipic acid (99%), diethyl ethoxymethylenemalonate (DEEM, 99%), L-fucose (>99%), L-arabinose (99%), L-glucose (99%), D-xylose 8>99%), D-mannose (>99%), D-mannitol (>98%), ferrous sulfate eptahydrate (>99%), potassium dichromate (0.1 N), sodium hydroxide (>97%), citric acid (>99.5%), ammonium formate (LC-MS grade), trifluoroacetic acid (>99%), potassium chloride (>99%), ascorbic acid (99%), sodium acetate (>99%), sulfuric acid (95.98%), hydrogen peroxide solution (30% *w/w*), phenol (99%), hydrochloric acid solution (37% *w/w*) chloride, phosphotungstic acid hydrate (99.995%), phosphomolybdic acid hydrate (99.99%), and Folin–Ciocalteu reagent (2N) were purchased from Merck (Italy). The deuterated solvents CD_3_OD and D_2_O were purchased from Sigma-Aldrich.

### 2.2. Sample Harvesting and Preparation

Samples of living PO (whole plant composed of roots, rhizomes, and leaves) were collected at the seabed of Bari in the Mediterranean Sea (with the authorisation of the local authorities), while the residues of PO were found along the shorelines. The samples were immediately transported to the laboratory, washed with tap water to remove salt and sand, and then by distilled water, and stored at 4 °C until their use. Samples of AN, ground and frozen, were harvested from Norwegian coasts and supplied by Valagro S.p.A., Chieti (CH), Italy. Fresh PO samples were divided into two parts, the leaves and the roots (including rhizomes), and analysed separately. Representative aliquots of each sample were oven-dried at 38 °C and finely ground with a PULVERISETTE 14 cutting mill equipped with 0.08–6 mm sieve rings, before running chemical and spectroscopic analyses and before the extraction procedure. The analyses of each analytical parameter were carried out in three replicates.

### 2.3. Chemical Characterization

The pH_H2O_ was determined by an EcoScan pH Meter and combined glass electrode, suspending 3 g of sample in 50 mL of distilled water. The suspension was stirred and left to rest for 12 h before measuring. Before carrying out the analyses, the residual humidity was determined by oven heating at 105 °C, to report the analytical results to the dry substance. The ash content, expressed as a percentage of the dry weight, was determined by combustion in a muffle at 550 °C for about 12 h. Total N (N_tot_) was determined according to the Kjeldahl method [18] whereas NH_3_-N was determined on the ground sample by extraction with 1 N KCl and subsequent extract distillation following the previous method. The organic carbon (OC) was determined by dichromate oxidation at defined conditions of acidity (20 mL H_2_SO_4_) and temperature (160 °C) and titration with 0.2 N ferrous sulfate, in the presence of 4-di-phenylamine sulfonate of barium or sodium as an indicator [19]. The content of the main macro- and microelements was determined by optical plasma emission spectroscopy (ICP-OES), after mineralization with 5 mL of HCl 0.5 M and 10 mL of H_2_O_2_ at 30% in a microwave and by filtration with Whatman 42 filters.

### 2.4. Extraction

All samples (PO leaves, PO roots, PO residues, and AN) were subjected to four different extraction protocols (in acid, alkaline, hydroalcoholic, and alcoholic solutions) (Table 1). Extraction temperatures and times were chosen based on the literature and previous experiments on the development of extraction methods in order to obtain the highest yield in terms of biomolecules [20,21,22]. For acid extraction (1), 5 g of sample was dissolved in 100 mL of extracting solution (1:20) and incubated for 4 h at 40 °C in a stirring thermostatic water bath. The extracting solution was prepared by dissolving 3.6 g of citric acid (0.6% C_6_H_8_O_7_) and 1.8 g of ascorbic acid (0.3% C_6_H_8_O_6_) in 600 mL of distilled H_2_O. For the alkaline extraction (2), 5 g of sample was dissolved in 100 mL of 1.5M NaOH solution (1:20) and incubated for 4 h at 80 °C in a stirring thermostatic water bath. After the incubation, the acid and alkaline extracts were filtered with a filter sock and centrifuged at 5000 rpm for 10 min. The liquid extracts were freeze-dried for subsequent analyses. For the hydroalcoholic (3) and alcoholic (4) extractions, 5 g of sample was dissolved in 100 mL of methanol solution (50% and 100%, respectively) (1:20) and incubated for 1.5 h at 40 °C under agitation. The samples were subsequently sonicated for 30 min and re-incubated at 40 °C for 30 min while stirring. The liquid extract was finally filtered with a 0.45 µm syringe filter and stored at −20 °C.

### 2.5. Carbohydrate Composition

The total carbohydrate content and sugar composition of Extracts 1 and 2 were determined after acidic hydrolysis, with the TFA at 120 °C × 1 h [23]. The monosaccharides were analysed using high-performance anion-exchange chromatography with pulsed amperometric detection (HPAEC-PAD, Dionex ICS 6000, ED40 Electrochemical Detector) equipped with a Thermo Fisher Scientific Dionex CarboPac PA20 column (3 × 150 mm) coupled with a guard column (3 × 30 mm). Samples were filtered (0.2 Ny) before analysis and injected with a Dionex AS-AP autosampler. The eluent flow rate was 0.400 mL/min and the temperature was kept at 30 °C. The mobile phase was made of the following A, H_2_O Milli-Q; B, 0.2 M sodium hydroxide; and C, 0.1 M sodium hydroxide, 0.1 M sodium acetate. Elution was performed following the following method: 0–21 min, 1.5% B; 21–33 min, 50% B; 33–49 min, 100% C; 49–53 min, 100% A; and 53–70 min, 1.5% B [24]. A standard solution of adonitol, mannitol, fucose, arabinose, galactose, glucose, xylose, and mannose was prepared to dissolve 25 mg of each sugar standard in 50 mL of Milli-Q water. Five scalar dilutions of standard solution in a 1–0.0625 ppm range were prepared for calibration. All experiments were carried out in triplicate and results were expressed on a dry weight percentage basis. Data were processed with the Dionex Chromeleon software.

### 2.6. Aminoacidic Composition

Separation and quantification of the amino acids in Extracts 1 and 2 were performed using high-performance liquid chromatography (HPLC) equipped with a UV–visible detector, following hydrolysis with 6 N HCl 0.1% phenol at 110 °C × 24 h under magnetic stirring (500 rpm) as described in [25] with minor modifications. During the hydrolysis, tryptophan is destroyed while asparagine and glutamine are deamidated to the corresponding aspartic acid and glutamic acid. Consequently, the quantification was limited to 17 amino acids. After the hydrolysis, samples were neutralized with 6 N sodium hydroxide and subjected to a reaction of derivatization with diethylethoxymethylenemalonate (DEEMM), which reacts with amino acids to form aminoenone derivatives absorbing at the wavelength of 280 nm. The derivatization was performed according to the methods in [26], with some modifications. The reacting mixture included 1 mL of 1 M borate buffer (pH 9.0), 500 µL of MeOH, 100 µL of internal standard (2-aminoadipic acid, 100 mg/L), 1 mL of sample, and 50 µL of DEEMM. The mixture was then heated at 70 °C for 2 h to allow for the complete degradation of excess DEEMM and other bioproducts. A standard solution was prepared to dissolve 25 mg of each amino acid standard in 25 mL of 0.1 N HCl. Five scalar dilutions of the standard in a 100–0.16 ppm range were prepared and underwent the same process of derivatization as the samples. The target compounds were identified and quantified according to the retention times and using the calibration curves of their corresponding standards. For chromatographic analysis, an Agilent 1200 Series HPLC system was used with a Waters Cortecs^®^ BEH particle size of 2.7 µm (2.1 mm × 150 mm) in a C18 analytical column equipped with a guard column Waters BEH 3 × 30 mm. The detection wavelength of the UV detector was 280 nm bandwidth 4 nm; the column temperature was maintained at 30 °C, the flow rate was 0.3 mL/min, and the injection volume was 2 µL. The elution solvents were 25 mM ammonium formate 5% acetonitrile (A) and acetonitrile 25 mM ammonium formate (70:30) (B) with the following gradient program: 0–5 min, 100% A; 5–33 min 50% A:50% B; 33–38 min, 20% A:80% B; and 38–50 min 100%A.

### 2.7. Total Phenolic Content

The determination of the total content of phenolic compounds was carried out with the Folin–Ciocalteu method. The Folin–Ciocalteu reagent, a mixture of phosphotungstic acid (H_3_PW_12_O_40_) and phosphomolybdic acid (H_3_PMo_12_O_40_), in the presence of phenols, is reduced to a mixture of tungsten and molybdenum oxides (W_8_O_23_ and Mo_8_O_23_) and a blue colour develops. The blue colour intensity can be read, using a UV–visible spectrophotometer, at a wavelength of 750 nm. By convention, the phenolic content was expressed in µg/g of gallic acid. In particular, 4.9 mL of H_2_O and 0.5 mL of Folin reagent were added to 100 µL of methanol extract (Extracts 3 and 4) and suitably diluted; after stirring for 3 min, 1.5 mL of 20% Na_2_CO_3_ was added and the samples were incubated in a bath thermostated at 40 °C for 20 min. At last, the solution was brought to a final volume of 10 mL with H_2_O and the absorbance was measured at 750 nm in 1 cm cuvettes using a Varian Cary 50 ScanUV–Visible Spectrophotometer. For the preparation of the calibration curve, scalar dilutions of the methanolic solution of gallic acid in a 1.6–0.2 ppm range were prepared [27,28].

### 2.8. NMR Analysis

The molecular composition of the obtained extracts was analysed by NMR spectroscopy and, in particular, by ^1^H-NMR, ^1^H-^1^H COrrelated Spectroscopy (COSY), and Diffusion-Ordered SpectroscopY (DOSY). NMR experiments were performed on a 14.1 T Bruker AVANCE III 600 MHz NMR spectrometer operating at the ^1^H frequency of 600.13 MHz and equipped with a TXI CryoProbe. Standard pulse sequences from the Bruker library were used for the experiments, and acquisition and processing were performed using Bruker’s TopSpin software. For each sample, 1D ^1^H spectra were recorded with the following parameters: number of scans, 8–256, depending on the concentration of the components in the sample; spectral width, 6009 Hz; 90° pulse width, 5.0 μs; relaxation delay, 5 s; acquisition time, 1.36 s; and temperature, 300 K. Post-acquisition phase adjustments and baseline corrections were applied before the manual integration of the peaks. For the DOSY experiments, an echo pulse sequence with bipolar gradients was used; the pulsed gradient (δ, 2 ms) strength was logarithmically increased in 32 steps, from 5% up to 95% of the maximum strength, with a diffusion time (Δ) of 200 ms. Pseudo-2D DOSY plots provide a direct visualization of signals belonging to different molecules in a complex mixture, spreading out the peaks in a second dimension according to the diffusion coefficients of the respective molecules. As the diffusion coefficient is inversely proportional to the hydrodynamic radius, according to the Stokes–Einstein equation, higher diffusion coefficients correspond in general to smaller molecules. Chemical shifts are expressed in ppm relative and referenced to the residual protonated water and methanol signals (4.79 ppm and 3.34 ppm, respectively). The methanolic extracts were concentrated under a vacuum by a rotary evaporator and redissolved in deuterated solvents (CD_3_OD or CD_3_OD/D_2_O) to avoid large solvent signals in the spectrum. All samples were prepared by dissolving about 10 mg of powder in 600 µL of deuterated solvent and transferred to a 5 mm NMR tube.

### 2.9. Statistical Analysis

The carbohydrate, aminoacidic, and total polyphenolic content were analysed by means of principal component analysis [29], after centring and scaling to unit variance, using MATLAB 2019b (MathWorks, Inc., Natick, MA, USA). The PCA model up to three principal components described 77% of the total variance in the data.

## 3. Results and Discussions

From the PO, both the dead fraction (coastal residues) and the living plant (sea) were taken, and the latter was divided into two parts, roots (including rhizomes) and leaves, which were analysed separately. For the AN, only seaweed from the sea was used for analyses. Therefore, in total, four different materials were studied (PO residues, PO roots, PO leaves, and AN). The fractions of all four materials were exposed to four different extraction procedures (acid, alkaline, hydroalcoholic, and alcoholic extraction), resulting in a large (4 × 4) sample matrix. The aqueous extracts were used for the quantification of carbohydrates and amino acids by ion chromatography and HPLC analysis, while the methanol extracts were subjected to the dosage of polyphenols using the Folin–Ciocalteu method. All extracts were subjected to 1D and 2D NMR spectroscopic analysis.

### 3.1. Chemical Features

The characterization of PO showed a greater ash content (inorganic components) in the PO residues compared to the fresh plant, proving that the PO residues, once stranded, tend to lose the organic component, e.g., due to fermentation processes and the concomitant release of CO_2_. This was confirmed by the values of organic carbon and total nitrogen (Table 2). Considering the PO fresh plant (roots and leaves), most macro- and microelement contents were greater than in the PO residues and this is probably due to, e.g., the rain-related leaching effect of the PO washed up on the beach. The only microelements that were more concentrated in the PO residues were Fe and Mo, confirming previous results [30]. Comparing the different parts of the PO fresh plant, micro- and macro elements appeared to be separated according to the plant portions. In particular, the leaves tended to accumulate more B, Mn, Zn, Ca, and Mg while the roots showed more Fe, Mo, Cu, Na, and K, in agreement with the literature [30,31,32]. The PO, both beached and fresh, showed in general higher contents of macro- and microelements compared to the AN, with the visible exception of K and Zn, showing an almost threefold higher concentration than in the PO residues. Among the microelements, the most abundant in both species were Fe and B.

### 3.2. Bio(macro)molecules

All the results relating to the carbohydrate and amino acid composition and the total phenolic content of the PO and AN extracts are shown in Table 3. The study of carbohydrates and amino acids was carried out on aqueous extracts (Extract 1: acid extraction and Extract 2: alkaline extraction) as they are usually richer in these molecules and because they are usually the preferred extracts for possible agricultural applications. Six well-separated carbohydrate peaks were identified in each chromatogram. The carbohydrate extraction yields appear to be more effective in the alkaline medium for PO residues and PO roots; conversely, the acidic extraction is more promising for PO leaves and AN. AN is extremely rich in carbohydrates with a total content of 14.0% DW, compared to living *Posidonia oceanica* with about 7% DW (whole plant, sum of roots, and leaves). In the seaweed AN, the main identified monosaccharide was mannitol which represented about 50% of the total content (~7%), followed by fucose (Fuc), glucose (Gluc), and xylose (Xyl). Galactose (Gal) and mannose (Man) were minor components. In the PO, the highest carbohydrate content was found in the roots with Gluc, Xyl, Gal, and arabinose (Ara) as the main components, with low concentrations of Fuc and Man, and a complete absence of mannitol. This profile is in correspondence with previous research [33]. PO leaves are characterized by a low carbohydrate content and as expected, compared to the fresh plant, the PO residues lost most of the organic component (minus 80% of carbohydrates) with a total content between 0.7 and 1.2% in the acid and alkaline extracts, respectively. This result agrees with what was obtained in [34]. This could be due to abiotic leaching phenomena of water-soluble monomeric carbohydrates after the cell wall rupture of dead leaves in *Posidonia* material [35].

From the results of the amino acid analysis, the acid extraction was found to be more efficient for the fresh plant of PO, whilst for the residues of PO and AN, the best extraction procedure seems to have been the alkaline one. Unlike the carbohydrate content, the fresh PO had a higher aminoacidic content than AN (29.5% of the whole plant of PO in the acid extracts, compared to about 6% of the AN alkaline extract). The concentration of aspartic acid (Asp) and glutamic acid (Glu) was the highest in all the samples, in agreement with the results in [36], followed by arginine (Arg), cysteine (Cys), and alanine (Ala) in the PO roots and leucine (Leu), lysine (Lys), and Ala in the PO leaves and AN, while generally, methionine (Met), threonine (Thr), histidine (His), and serine (Ser) were the lowest. Arg was absent in the PO leaves and AN, while in the PO roots, Ser and isoleucine (Ileu) were not found. The alkaline solution efficiently extracted the amino acid component of the PO residues with a total content of 4.1%, which was lower than the fresh plant but in line with the results obtained for AN. The amino acid proline (Pro) was not identifiable because, due to its secondary amide group, it absorbs at a different wavelength (292 nm) [26], while the amino acid tyrosine (Tyr) was not indicated because the peak coeluted with a residual peak of the derivatizing agent. The analysis of the total phenolic content (TPC) was carried out on the alcoholic extracts (Extract 3: CH_3_OH 50% and Extract 4: CH_3_OH 100%) as the polyphenols are more soluble in organic solvents. AN had the highest content of polyphenols with 115 mgGAE/g DW in comparison with 48.2 mgGAE/g DW of the PO leaves and 47 mgGAE/g DW of the PO roots [37,38]. The PO residues appeared to have lost most of their polyphenols (about 90%), which is in line with expectation as these are very sensitive to degradation and oxidation, phenomena likely to occur in a dead plant left for a long time and deposited on the coast.

**Figure 1:**
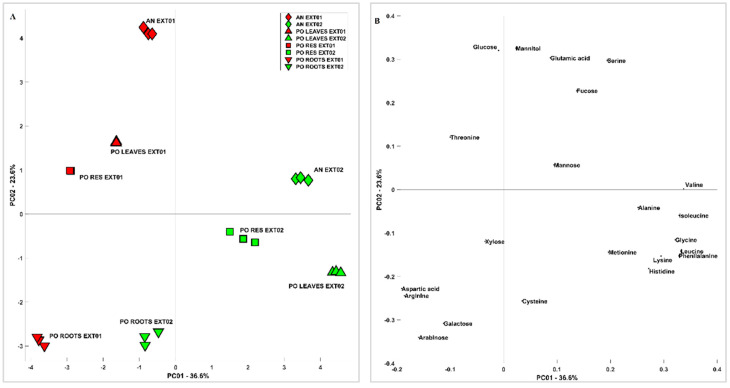
The PCA of the carbohydrate and aminoacidic content of *Posidonia oceanica* and *Ascophyllum nodosum* Extracts 1 and 2. (**A**) Scores and (**B**) loadings of PC1 vs. PC2.

**Figure 2:**
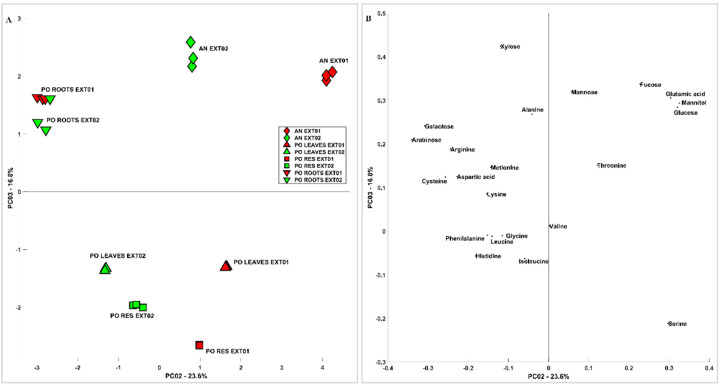
The PCA of the carbohydrate and aminoacidic content of *Posidonia oceanica* and *Ascophyllum nodosum* Extracts 1 and 2. (**A**) Scores and (**B**) loadings of PC2 vs. PC3.

In order to obtain a multivariate description of the similarities and differences among samples and extraction methods, carbohydrate and aminoacidic contents were analysed by means of principal component analysis (PCA). Figure 1 shows the scores (A) and loadings (B) of PC1 vs. PC2; the first component carries indications of the phenomenon that differentiates the samples the most, which is the method of extraction. Generally speaking, considering the variability related to PC1, extraction methods affect root samples less than other samples. Extraction Method 2 recovered more minor amino acids and shows more spread out results when the different samples are considered, while Extraction Method 1 recovered more the more abundant components of amino acids and carbohydrates. On PC2, the difference between AN and PO is clearer when considering Extraction Method 1, in particular related to sugars such as Gluc and mannitol and amino acids such as Glu and Asp; this difference is significantly lower when considering Extraction Method 2. Considering the scores (A) and loadings (B) of PC2 vs. PC3 reported in Figure 2, the differences among samples are more clear and less related to the extraction method, which influences mostly PC1. The PO roots, in particular, were richer in Ara, Gal, and amino acids such as Arg and Cys, while the leaves and residues were more similar to each other. The difference between AN and PO is still clear and related to most of the species already discussed.

### 3.3. 1D and 2D ^1^H-NMR Analyses of PO and AN

As described above, next to the AN seaweed, from the PO plant, three different samples were taken: the material washed ashore and the fresh sea plant material, from which the roots and leaves were separately analysed. All four samples were submitted to four different extraction procedures (acid, alkaline, alcoholic, and water/alcohol). All sample extracts were separately subjected to one-dimensional ^1^H-NMR and two-dimensional ^1^H-^1^H COrrelated SpectroscopY (COSY) and Diffusion-Ordered SpectroscopY (DOSY) analysis. Below, the first typical datasets of a representative sample are described, comprising the 1D, COSY, and DOSY spectra allowing for the assignment of a discrete number of peaks to specific metabolites. Consecutively, the assigned metabolites are listed and discussed, followed by a comparison of the AN and PO samples. Then, from the three different PO samples, the NMR analysis of the beached material is compared to the root and the leaf analysis. Finally, the four different extraction methods are compared.

#### 3.3.1. Molecular Characterization of *Posidonia oceanica* and *Ascophyllum nodosum*

**Figure 3:**
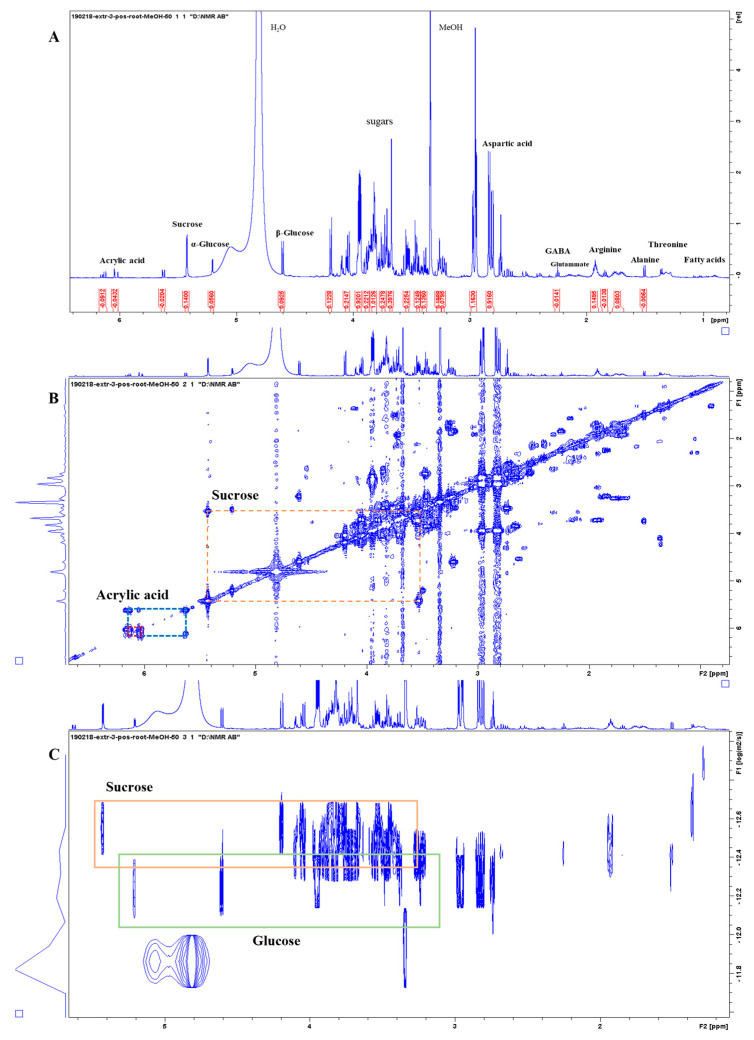
NMR spectra of *Posidonia oceanica* root extract (CH_3_OH 50% extraction). (**A**) The 1D ^1^H-NMR spectrum with a selection of metabolites highlighted; the aromatic regions are not shown here for clarity reasons and full spectra are provided in the Supplementary Materials. (**B**) The 2D ^1^H-^1^H-COSY NMR spectrum with representative off-diagonal cross peaks highlighted for sucrose and acrylic acid. (**C**) The 2D ^1^H-^1^H-DOSY NMR spectrum with the orange and green boxes highlighting the sucrose and glucose signals, respectively. The vertical axis in the pseudo-2D plot represents the diffusion coefficient of the molecules generating the signals; the monomeric glucose clearly has a much higher diffusion coefficient (i.e., is faster, smaller) than the dimeric sugar sucrose.

Figure 3 shows representative ^1^H-NMR spectra of the PO alcoholic extract (Extract 3) that allowed for the largest number of molecules to be identified in the PO. In the region between 0.0 and 3.0 ppm, the amino acids Asp (δ 2.8 (dd), 2.94 (dd), 3.93 (dd)), Glu (δ 2.05 (m), δ 2.4 (m)), Ala (doublet at δ 1.51), Arg (δ 1.7 (m), 1.93 (m), 3.26 (t), 3.74 (t)), Thr (doublet at δ 1.36), γ-aminobutyric acid (GABA) (δ 1.85 (m), 2.25 (t), 3.22 (t)), and some peaks related to fatty acids, in particular related to oleic and α-linoleic acid (C18: 2 Δ 9.12) (Face, 1993), were identified; in the central part of the spectrum (3.0–5.5 ppm), there were signals related to sugars. It was difficult to obtain all the individual signals of the different sugars as many of them were overlapping, but among them, specific characteristic peaks were identified such as those of Gluc (α- and β-gluc anomeric hydrogens at δ 5.21 and 4.61) and sucrose (Suc) (Gluc and fructose anomeric hydrogens at δ 5.42 and 3.67). In particular, with the help of the DOSY NMR, it was possible to distinguish monosaccharide from disaccharide signals, such as Gluc and Suc, as they are separated based on a different diffusion coefficient related to their considerably different sizes (Figure 3C). In the low-field region, in addition to several peaks related to aromatic compounds, there are the signals for acrylic acid. The aromatic region (5.5–10.0 ppm) was particularly rich in peaks, especially in the PO roots and leaves, but the spectra were difficult to interpret and not further considered here.

**Figure 4:**
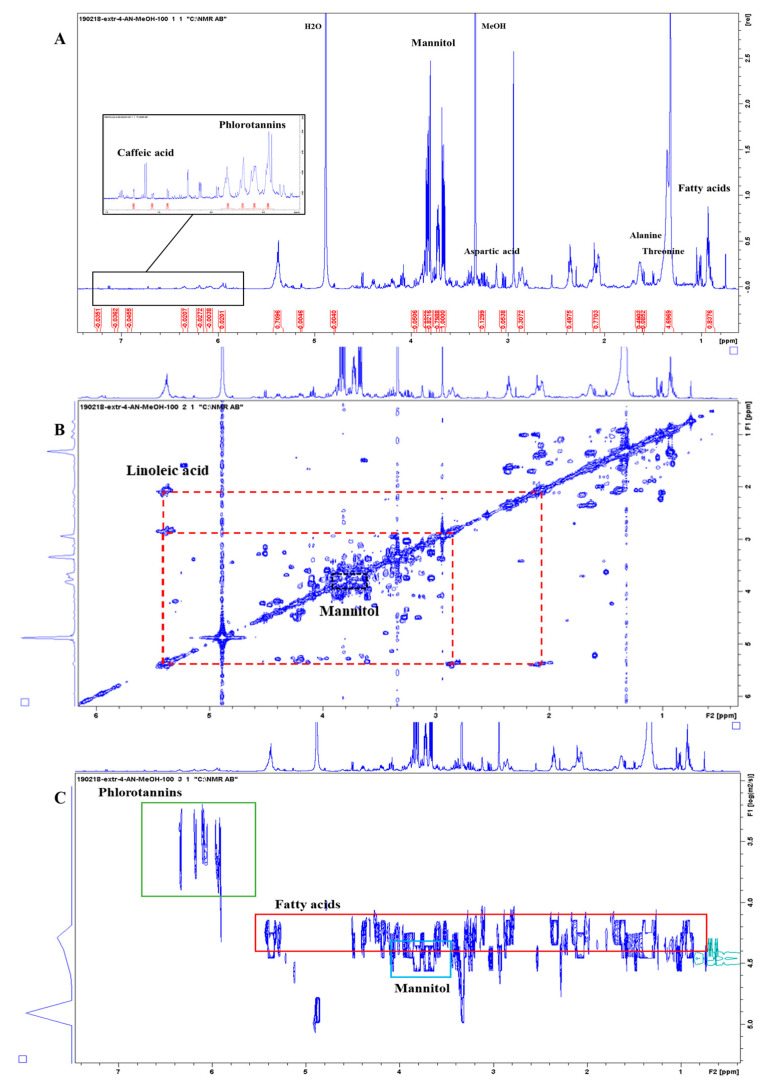
NMR spectra of *Ascophyllum nodosum* extract (CH_3_OH extraction). (**A**) The 1D ^1^H-NMR spectrum with a selection of metabolites highlighted. The aromatic regions are not shown here for clarity reasons and full spectra are provided in the Supplementary Materials. (**B**) The 2D ^1^H-^1^H-COSY NMR spectrum with representative off-diagonal cross peaks indicated for mannitol and linoleic acid. (**C**) The 2D ^1^H-^1^H-DOSY NMR spectrum with the green, red, and blue boxes highlighting the phlorotannins, fatty acids, and mannitol signals, respectively, separated according to their different diffusion coefficients.

Considering the samples of *Ascophyllum nodosum*, compounds identified in the alcoholic extract (Extract 4) comprised mannitol, α and β-Gluc (low concentration), the amino acids Glu, Ala, and Thr, fatty acids including oleic (C18:1 Δ 9) and linoleic acid (C18: 2 Δ 9,12), as well as phlorotannins (Figure 4). Mannitol is an important osmoprotectant as it gives the seaweed greater tolerability to abiotic stresses such as increased salinity [39]. It also acts as a chelating agent [40]. Phlorotannins are polymers of phloroglucinol, characteristic of brown algae, which because of the numerous phenolic rings present in their structure, have a very strong antioxidant power [41,42]. The lipophilic components of the AN extract seem to modulate the expression of the genes involved in cold response resulting in an enhanced tolerance to freezing temperatures [43,44]. There are still several signals to assign but the overlap and lack of data in the literature makes the interpretation cumbersome. Complementary strategies could be used to increase the number of identified molecules, e.g., by using total correlation NMR experiments, TOCSYs, or by exploiting heteronuclear 2D NMRs such as ^1^H-^13^C-HSQC (short-range) and HMBC (long-range) experiments. This is however beyond the scope of the current paper.

#### 3.3.2. Metabolite Identification of *Posidonia oceanica* and *Ascophyllum nodosum*

The ^1^H-NMR spectra can be roughly subdivided into three main regions: a high-field region (0.0–3.0 ppm) that collects the amino acid (back bone and aliphatic side groups) and lipid signals; a medium-field region (3.0–5.5 ppm) that contains the sugar signals; and a low-field region (5.5–10.0 ppm) dominated by aromatic compounds, organic acids, and polyphenols, and also the signals from amino acids with aromatic side chain groups such as phenylalanine, histidine, tryptophan, or tyrosine. In cases of overlapping peaks, 2D (COSY) and pseudo-2D (DOSY) data were consulted (see previous section) to correlate specific signals and allow for their assignment to specific molecules. The assignment of the peaks in the ^1^H-NMR spectra was performed by consulting the literature [45,46,47] and the database sources Biological Magnetic Resonance Data Bank (BMRB) and Human Metabolome Database (HMDB) [48,49]. The list of metabolites identified in the two species is reported in Table 4 (chemical shifts are sometimes slightly shifted with respect to values in the literature due to the extraction conditions).

#### 3.3.3. Comparison between *Posidonia oceanica* Roots, Leaves, and Residues

**Figure 5:**
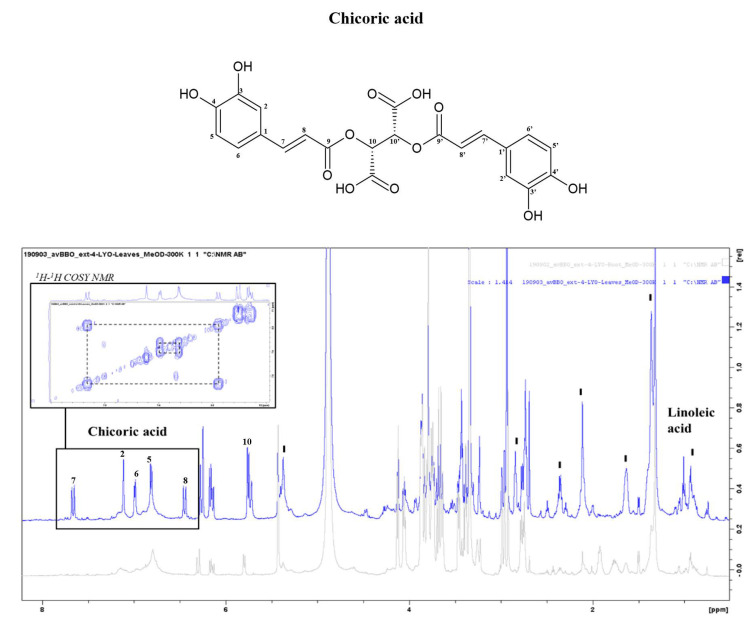
NMR spectra of *Posidonia oceanica* leaf extracts (MeOH 100% extraction) (blue signal) in comparison with root extracts (grey signal).

The profile of the PO roots, presented in the paragraph3.3.1 (Figure 3), is common to all parts of the PO with some differences: in the leaf samples, there were no GABA and Arg signals, in accordance with the results obtained from the chemical analysis, but at the same time, in the aromatic region, six signals related to the polyphenol chicoric acid were evident (Figure 5), in accordance with results by others [50,51]. It is a tartaric acid ester of two caffeic acids typically present in the leaves of the chicory plant. The presence of chicoric acid is a good indicator of the freshness of the plant as numerous studies have shown that its concentration in the leaves increases according to the youth of the plant [52]. This phenol has important properties such as antioxidant and antibacterial activity and health benefits such as anti-diabetic, antiviral, and anti-cancer activity. For example, chicoric acid interferes with the replication of human immunodeficiency virus type 1 (HIV-1), inhibiting the HIV-1 integrase enzyme [53]. Furthermore, in the PO leaf spectrum, the peaks of linoleic fatty acids were very evident [43,54].

**Figure 6:**
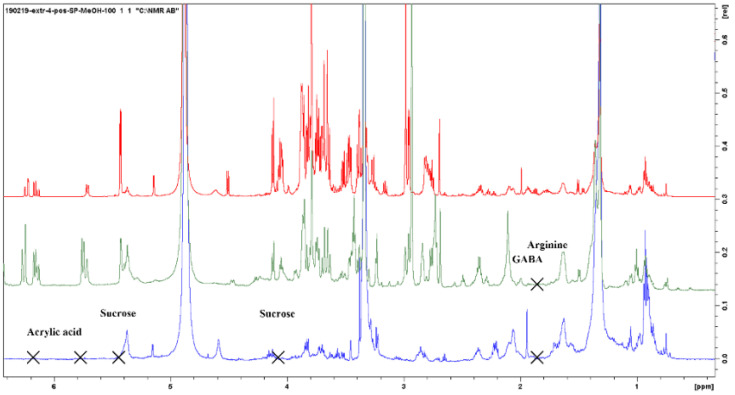
Comparison of 1D NMR spectra of alcoholic extracts of roots (CH_3_OH 100%) (red signal), leaves (green signal), and residues (blue signal) of *Posidonia oceanica*.

The PO residue spectra generally show fewer peaks than the fresh PO and in particular, the signals of Asp, Arg, GABA, Suc, and acrylic acid are missing or are poorly visible (Figure 6). This is quite understandable considering that it is a washed ashore remnant of the plant. On the other hand, the signals of Glu, Ala, Thr, linoleic acid, and chicoric acid are clearly visible, and there are traces of other molecules present at very low concentrations which have not been further identified.

#### 3.3.4. Evaluation of Extraction Methods

**Figure 7:**
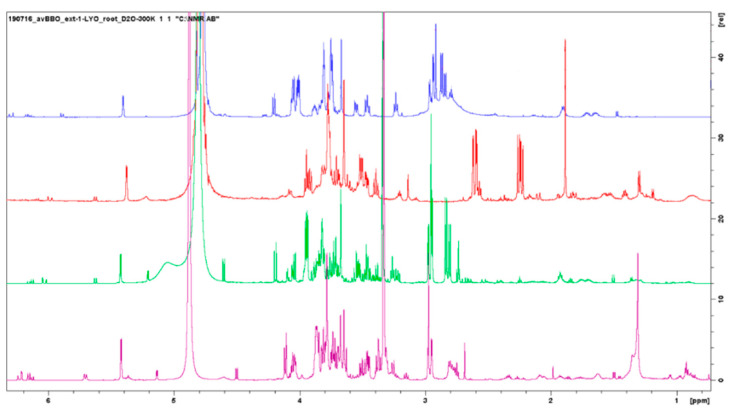
Comparison between 4 types of *Posidonia oceanica* roots extracts: acid extract (blue signal), alkaline extract (red signal), CH_3_OH 50% extract (green signal), and CH_3_OH 100% extract (purple signal).

A direct comparison of the samples obtained by the different extraction methods was complicated as the peaks of the same molecule could show different chemical shifts depending on the extraction conditions used. Samples obtained by alkaline extraction, for example, showed, in general, peaks shifted more to the right (lower δ) compared to the neutral or acid pH (Figure 7). What we can observe clearly from the NMR spectra is that those of the alcoholic extracts are characterized by a greater number of peaks and they allowed us to study the presence of different categories of biomolecules. In particular, alcoholic extracts allow for appreciating the presence of fatty acids and polyphenols scarcely present in aqueous extracts. However, if the extracts are to be used for agricultural purposes and an industrial production is planned, the use of methanol as an extraction solvent would make the method more expensive and disadvantageous for the necessary purification procedures and removal of the extraction solvent; for this reason, one could think of optimizing the aqueous extraction methods to obtain a greater number of extracted molecules.

## 4. Conclusions

*Posidonia oceanica* and *Ascophyllum nodosum* are characterized by a great variety of bio(macro)molecules. In addition to their richness in micro- and macroelements, both species show a high content of carbohydrates, amino acids, and phenols. In particular, PO is characterized by relatively high quantities of glucose, xylose, galactose, and arabinose, aspartic acid, polyunsaturated fatty acids, and the phenol chicoric acid. The roots and rhizomes of live PO are richer in carbohydrates and amino acids than the leaves are, which is not surprising considering the fact that rhizomes represent a reserve tissue for the plant. At the same time, the leaves could have an important potential as they are rich in polyphenols including, in particular, chicoric acid and important microelements such as B, Ca, and Zn. The seaweed AN is characterized, instead, predominantly by the presence of mannitol, glutamic acid, polyunsaturated fatty acids, and phlorotannins. Generally, PO shows a relatively higher content of amino acids, while AN has higher amounts of carbohydrates. The analysis of the PO residues showed that the plant, even if dead, still contained large quantities of inorganic and organic material of biochemical importance. The PO residues were particularly rich in Ca, B, Fe, Mo, and Mn, maintaining rather high levels of organic C and N. Furthermore, even if at low concentrations, the PO residues contained most of the amino acids and sugars and their composition did not differ much from the fresh leaves. This research encourages further investigations into the possible uses of PO and NA extracts or even the plant (alive or beached)/seaweed as such, which are rich in bioactive molecules, for example, for the fertilization of agricultural fields. In particular, the possibility of reusing the PO residues is in line with the exploitation of environmentally friendly materials through an economically sustainable approach. The recycling and valorisation of waste biomass has great environmental and economic value as it reduces the quantity of waste and allows for the recovery of raw materials and energy. Furthermore, this study shows an evaluation of four different extraction procedures that, on the one hand, provide distinct sets of metabolite spectra, allowing for a more in-depth metabolomic profiling. On the other hand, these extractions show specific and different concentrations of different metabolites, of interest as possible formulations for further use. They might need further optimization to obtain a higher yield in biomolecules if they are to be used for some specific purpose. Although the analysis of the NMR spectra of mixtures of compounds such as extracts of marine algae or plants is complex, the application of this technique to the study of the metabolome of plants shows great potential for quickly obtaining information on the profiles of biomolecules in plant species without cumbersome pre-treatments required. This is important not only for an immediate and direct comparison of different species, but also for the comparison of the same species under different environmental conditions (e.g., different extractions, fresh plants collected in the sea, dead plants washed ashore on the coast). Therefore, NMR spectroscopy is a most promising tool for the construction of molecular fingerprints of plant species. Here, only 1D ^1^H NMR, ^1^H-^1^H COSY, and DOSY spectra were used, but the arsenal of NMR pulse sequences is vast and metabolomic profiling can be extended, e.g., by total correlation experimentation, TOCSY, or the exploitation of heteronuclear 2D NMR such as ^1^H-^13^C-HSQC (short-range) and HMBC (long-range) experiments.

## Figures and Tables

**Table 1:** Extraction Methods.

Extractions			
No. 1 (Acid Extraction)	No. 2 (Alkaline Extraction)	No. 3 (Hydroalcoholic Extraction)	No. 4 (Alcoholic Extraction)
Citric acid (0.6%), ascorbic acid (0.3%)	NaOH 1.5 M	Methanol 50%	Methanol 100%
40 °C × 4 h (shaker water bath)	80 °C × 4 h (shaker water bath)	40 °C × 1.5 h (shaker water bath)	40 °C × 1.5 h (shaker water bath)
Filtration	Filtration	30 min sonication	30 min sonication
Centrifugation: 5000 rpm × 10 min	Centrifugation: 5000 rpm × 10 min	30 min, 40 °C (shaker water bath)	30 min, 40 °C (shaker water bath)
		Filtration with syringe filter 0.45 µm	Filtration with syringe filter 0.45 µm

**Table 2:** Chemical compositions of residues and fresh plants (divided into leaves and roots) of *Posidonia oceanica* and *Ascophyllum nodosum*.

Chemical Features (Mean ± Standard Deviation)					
		*Posidonia oceanica*			*Ascophyllum nodosum*
		Residues	Fresh		
			ROOTS	LEAVES	
**pH_H2O_**		7.9 ± 0.1	7.9 ± 0.2	7.7 ± 0.1	6.3 ± 0.1
**Ash**	(%)	28.2 ± 0.3	17.9 ± 0.2	25.8 ± 0.1	18 ± 0.5
**N_tot_**	(g kg-1 d.m.)	5.7 ± 0.2	14.8 ± 0.7	14.8 ± 0.02	12.3 ± 0.4
**N-NH_4_**	(g kg-1 d.m.)	0.12 ± 0.02	0.1 ± 0.01	0.08 ± 0.01	0.3 ± 0.01
**N_org_**	(g kg-1 d.m.)	5.58 ± 0.2	14.7 ± 0.7	14.72 ± 0.02	12 ± 0.4
**C_org_**	(g kg-1 d.m.)	359 ± 9	410.4 ± 14	378 ± 8	409.6 ± 12
**C/N**		63	27.7	25	32.5
**P_tot_**	(g kg-1 d.m.)	0.3 ± 0.01	0.34 ± 0.01	0.6 ± 0.02	0.61 ± 0.01
**Ca_tot_**	(g kg-1 d.m.)	33.4 ± 0.6	15.9 ± 0.2	45.8 ± 1	8 ± 0.05
**K_tot_**	(g kg-1 d.m.)	2.1 ± 0.01	4.7 ± 0.02	1.6 ± 0.01	11.4 ± 0.03
**Mg_tot_**	(g kg-1 d.m.)	7 ± 0.08	5 ± 0.05	10 ± 0.1	6 ± 0.01
**Na_tot_**	(g kg-1 d.m.)	20.7 ± 0.2	15.2 ± 0.2	4 ± 0.1	17 ± 0.1
**B_tot_**	(mg kg-1 d.m.)	1613.4 ± 24	852.3 ± 17	1427.9 ± 22	95 ± 1.6
**Fe_tot_**	(mg kg-1 d.m.)	4758 ± 68	2438.4 ± 36	489.9 ± 9	372.8 ± 8
**Mn_tot_**	(mg kg-1 d.m.)	130.1 ± 1.2	108.9 ± 1	369.3 ± 3.1	48.2 ± 0.6
**Mo_tot_**	(mg kg-1 d.m.)	36.2 ± 0.8	7 ± 0.2	0.2 ± 0.01	1.3 ± 0.01
**Zn_tot_**	(mg kg-1 d.m.)	37.3 ± 0.1	51.6 ± 0.1	113 ± 0.2	100.2 ± 0.22
**Cu_tot_**	(mg kg-1 d.m.)	28 ± 0.1	33 ± 0.1	22.2 ± 0.1	10 ± 0.05

**Table 3:** Carbohydrate, amino acid, and total phenolic content of *Posidonia oceanica* (residues and fresh plant, divided into leaves and roots) and *Ascophyllum nodosum* (results of carbohydrates and amino acids are expressed as percentages of dry weight; nd: not detected; rt: retention time).

			*Posidonia oceanica*						*Ascophyllum nodosum*	
			Residues		Fresh Plant					
					ROOTS		LEAVES			
**Carbohydrates (%)**										
			EXT 1	EXT 2	EXT 1	EXT 2	EXT 1	EXT 2	EXT 1	EXT 2
components		rt								
1	**Mannitol**	2.45	0	0	0	0	0	0	7.05 ± 0.25	2.51 ± 0.15
2	**Fucose**	4.27	0.06 ± 0.00	0.46 ± 0.02	0.05 ± 0.00	0.20 ± 0.01	0	0	2.00 ± 0.03	2.94 ± 0.12
3	**Arabinose**	8.6	0	0.17 ± 0.01	1.38 ± 0.01	1.14 ± 0.05	0.02 ± 0.01	0.08 ± 0.01	0	0
4	**Galactose**	10.5	0.16 ± 0.00	0.26 ± 0.01	1.52 ± 0.03	2.39 ± 0.39	0.12 ± 0.00	0.20 ± 0.00	0.24 ± 0.01	0.38 ± 0.01
5	**Glucose**	12.2	0.10 ± 0.00	0.12 ± 0.00	0.26 ± 0.01	1.23 ± 0.14	1.84 ± 0.04	0.44 ± 0.02	3.98 ± 0.04	1.54 ± 0.06
6	**Xylose**	14.4	0.24 ± 0.01	0.1± 0.00	1.65 ± 0.01	0.80 ± 0.14	0.03 ± 0.00	0.05 ± 0.02	0.57 ± 0.01	1.72 ± 0.07
7	**Mannose**	15.3	0.11 ± 0.00	0.09± 0.00	0.06 ± 0.01	0.24 ± 0.11	0	0	0.18 ± 0.02	0.47 ± 0.04
	**Tot**		**0.68** ± 0.02	**1.21** ± 0.04	**4.93** ± 0.04	**6.01** ± 0.19	**2.01** ± 0.04	**0.77** ± 0.06	**14.02** ± 0.35	**9.55** ± 0.44
**Amino acids (%)**										
components		rt								
1	**Aspartic acid**	2.78	0.31 ± 0.00	0.51 ± 0.03	13.94 ± 0.10	4.36 ± 0.18	7.25 ± 0.03	2.44 ± 0.02	0.42 ± 0.01	0.73 ± 0.00
2	**Glutamic acid**	3.78	0.27 ± 0.00	0.48 ± 0.03	0.76 ± 0.01	0.51 ± 0.02	1.27 ± 0.01	1.05 ± 0.01	2.28 ± 0.06	1.65 ± 0.03
3	**Serine**	8.79	0.18 ± 0.00	0.23 ± 0.01	0	0	0.27 ± 0.00	0.27 ± 0.01	0.23 ± 0.00	0.23 ± 0.00
4	**Histidine**	12.3	0.21 ± 0.00	0.29 ± 0.01	0.20 ± 0.02	0.29 ± 0.01	0.21 ± 0.00	0.30 ± 0.01	0.22 ± 0.01	0.26 ± 0.01
5	**Glycine**	12.65	0	0.39 ± 0.03	0	0.35 ± 0.01	0.12 ± 0.01	0.51 ± 0.02	0.14 ± 0.00	0.35 ± 0.00
6	**Threonine**	13.87	0	0	0	0.23 ± 0.01	0.20 ± 0.00	0	0.20 ± 0.00	0
7	**Arginine**	15.69	0	0	3.67 ± 0.08	0.47 ± 0.01	0	0	0	0
8	**Alanine**	16.66	0	0.37 ± 0.02	0.39 ± 0.00	0.34 ± 0.01	0.31 ± 0.00	0.59 ± 0.02	0.44 ± 0.01	0.54 ± 0.01
9	**Tyrosine**	19.52	nd	nd	nd	nd	nd	nd	nd	nd
10	**Valine**	21.55	0	0.32 ± 0.01	0	0.24 ± 0.01	0.23 ± 0.00	0.48 ± 0.00	0.20 ± 0.00	0.39 ± 0.01
11	**Metionine**	22.1	0	0	0.05 ± 0.00	0	0	0.08 ± 0.00	0	0.07 ± 0.00
12	**Isoleucine**	24.31	0	0.24 ± 0.01	0	0	0	0.32 ± 0.01	0	0.28 ± 0.01
13	**Leucine**	24.87	0	0.37 ± 0.02	0	0.26 ± 0.02	0	0.65 ± 0.01	0	0.50 ± 0.00
14	**Phenilalanine**	25.31	0	0.30 ± 0.01	0	0.22 ± 0.00	0	0.45 ± 0.01	0	0.36 ± 0.00
15	**Cysteine**	26.9	0	0.19 ± 0.01	0.09 ± 0.01	1.39 ± 0.04	0	0.31 ± 0.00	0	0.17 ± 0.01
16	**Lysine**	28.57	0	0.41 ± 0.03	0.29 ± 0.00	0.33 ± 0.00	0.25 ± 0.00	0.63 ± 0.00	0.24 ± 0.01	0.44 ± 0.00
17	**Proline**		nd	nd	nd	nd	nd	nd	nd	nd
	**Tot**		**0.97** ± 0.08	**4.10** ± 0.25	**19.39** ± 0.19	**9.00** ± 0.29	**10.11** ± 0.08	**8.08** ± 0.14	**4.36** ± 0.22	**5.97** ± 0.10
**Total phenols (mgGAE/g DW)**										
			EXT 3	EXT 4	EXT 3	EXT 4	EXT 3	EXT 4	EXT 3	EXT 4
			**0.49** ± 0.01	**0.72** ± 0.01	**46.9** ± 0.19	**40.8** ± 0.55	**48.2** ± 0.67	**29.7** ± 0.18	**115** ± 0.8	**15.6** ± 0.18

**Table 4:** Summary of the metabolites identified in the 600 MHz ^1^H-NMR spectrum of the alcoholic extracts of *Posidonia oceanica* and *Ascophyllum nodosum*. * Chicoric acid: specifically identified in the leaves and in small quantities in the residues.

*Posidonia oceanica*				
Compounds	Assignment	δ ^1^H (ppm)	Multiplicity	Connectivity
*Carbohydrates*				
α-D-glucose (α-Gluc)	CH-4	3.39	m	
	CH-2	3.49	dd	
	CH_2_-6	3.73	m	
	CH-3	3.74	m	
	CH-5, CH_2_-6	3.83	m	
	CH-1	5.21	d	1–2
β-D-glucose (β-Gluc)	CH-2	3.22	dd	2–3
	CH-4	3.39	m	4–5
	CH-3,5	3.46	m	3–4, 5–6
	CH_2_-6	3.76	m	
	CH_2_-6	3.85	dd	
	CH-1	4.61	d	1–2
Sucrose (Suc)	CH-4	3.46	t	4–5
	CH-2	3.53	dd	2–3
	CH_2_-1′ (Fru)	3.67	s	
	CH-3	3.74	t	3–4
	CH_2_-6,6′	3.81	m	
	CH-5	3.85	dd	
	CH-5′	3.87	dd	
	CH-4′	4.05	t	4′–5′
	CH-3′	4.19	d	3′–4′
	CH-1 (Gluc)	5.42	d	1–2
*Organic acids*				
Acrylic acid	CH_2_-3	5.61	dd	3–3
	CH_2_-3	6.02	dd	
	CH-2	6.13	dd	2–3
*Amino acids*				
Alanine (Ala)	CH_3_-3	1.51	d	
	CH-2	3.75	q	2–3
Arginine (Arg)	CH_2_-4	1.7	m	4–3,5
	CH_2_-3	1.93	m	3–4,2
	CH_2_-5	3.26	t	
	CH_2_-2	3.71	t	
Aspartic acid (Asp)	CH-3	2.82	dd	3–3
	CH-3	2.96	dd	
	CH-2	3.95	dd	2–3
Aminobutyric acid (GABA)	CH_2_-3	1.85	m	3–2,4
	CH_2_-2	2.25	t	
	CH_2_-4	3.22	t	
Glutamic acid (Glu) *	CH_2_-6	2.05	m	
	CH_2_-6	2.15	m	6–7, 6–4
	CH_2_-7	2.4	m	
	CH_2_-4	3.72	dd	
Threonine (Thr)	CH-3	1.36	d	
	CH_3_-4	4.12	m	
*Lipids: fatty acids*				
α-linoleic acid (C18:2 Δ 9,12)	CH_3_-18	0.9	t	18–17
	CH_2_-4,5,6,7,15,16,17	1.32	m	4,7–3,8; 15,17–14,18
	CH_2_-3	1.58	m	3–2,4
	CH_2_-8,14	2.06	m	8–7,9;14–13,15
	CH_2_-2	2.17	t	
	CH_2_-11	2.78	t	11–10,12
	=CH-9,10,12,13	5.39	m	9,10–8,11; 12,13–11,14
Oleic acid (C18:1 Δ 9)	CH_3_-18	0.92	t	18–17
	CH_2_-4,5,6,7,12,13,14, 15,16,17	1.34	m	4,7–3,8; 12–11;17–18
	CH_2_-3	1.64	m	3–2,4
	CH_2_-8,11	2.09	m	8–7,9;11–10,12
	CH_2_-2	2.34	t	
	=CH-9,10	5.37	m	9,10–8,11;
*Phenols*				
Chicoric acid *	CH-10,10′	5.57	s	
	CH-8,8′	6.49	d	8–7
	CH-5,5′	6.97	d	5–6
	CH-6,6′	7.17	d	
	CH-2,2′	7.25	d	
	CH-7,7′	7.73	d	
***Ascophyllum nodosum***				
*Carbohydrates*				
Mannitol (Mann)	CH_2_-1	3.66	dd	11
	CH-2	3.72	m	2–3
	CH-3	3.8	d	
	CH_2_-1	3.83	dd	
α-D-glucose (α-Gluc)	CH-4		m	
	CH-2	3.39	dd	
	CH_2_-6		m	
	CH-3		m	
	CH-5, CH_2_-6		m	
	CH-1	5.14	d	1–2
β-D-glucose (β-Gluc)	CH-2	3.27	dd	2–3
	CH-4		m	4–5
	CH-3,5	3.59	m	3–4, 5–6
	CH_2_-6		m	
	CH_2_-6	3.95	dd	
	CH-1	4.5	d	1–2
*Amino acids*				
Alanine (Ala)	CH_3_-3	1.5		
	CH-2	3.62		3–4
Threonine (Thr)	CH-3	1.43	d	
	CH_3_-4	4.23	m	
Glutamic acid (Glu)	CH_2_-6	2.03	m	
	CH_2_-6	2.17	m	6–7, 6–4
	CH_2_-7	2.37	m	
	CH_2_-4	3.81	dd	
*Lipids: fatty acids*				
α-Linoleic acid (C18:2 Δ 9,12)	CH_3_-18	0.92	t	18–17
	CH_2_-4,5,6,7,15,16,17	1.35	m	4,7–3,8; 15,17–14,18
	CH_2_-3	1.63	m	3–2,4
	CH_2_-8,14	2.06	m	8–7,9;14–13,15
	CH_2_-2	2.35	t	
	CH_2_-11	2.81	t	11–10,12
	=CH-9,10,12,13	5.35	m	9,10–8,11; 12,13–11,14
Oleic acid (C18:1 Δ 9)	CH_3_-18	0.92	t	18-17
	CH_2_-4,5,6,7,12,13,14, 15,16,17	1.35	m	4,7–3,8; 12–11;17–18
	CH_2_-3	1.62	m	3–2,4
	CH_2_-8,11	2.06	m	8–7,9;11–10,12
	CH_2_-2	2.34	t	
	=CH-9,10	5.37	m	9,10–8,11;
*Phenols*				
Phlorotannin		5.94		
		6.08		
		6.19		
		6.35		
Caffeic acid	=CH-10	6.33	d	
	CH-3	6.91	d	3–4
	CH-4	7.05	dd	
	CH-6	7.15	d	
	=CH-9	7.24	d	4–9

## Data Availability

Data are contained within the article or Supplementary Materials.

## Supplementary Materials

The following supporting information can be downloaded at: https://www.mdpi.com/article/10.3390/metabo13020170/s1, Figure S1: NMR spectra of all extracts.
