# Supplementary material for: Chemical Composition, Biomolecular Analysis, and Nuclear Magnetic Resonance Spectroscopic Fingerprinting of Posidonia oceanica and Ascophyllum nodosum Extracts
Source: Metabolites. 2023 Jan 24;13(2):170. doi: 10.3390/metabo13020170 (PMC9963245; doi:10.3390/metabo13020170)
Supplement: Supplementary file 1 [file metabolites-13-00170-s001.zip › metabolites-2127635-supplementary.pdf]

ESTRAZIONE 1: FIG.1-3 PO ROOTS, FIG. 4-6 PO LEAVES, FIG.7-9 PO RESIDUES, FIG.10-12 AN

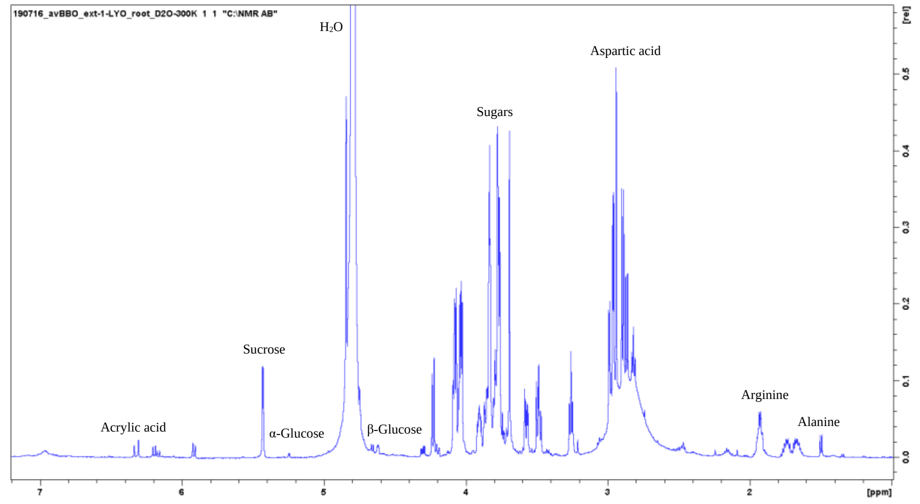

Figure S1 Extraction 1, 1D ^{1}H-NMR of PO Roots

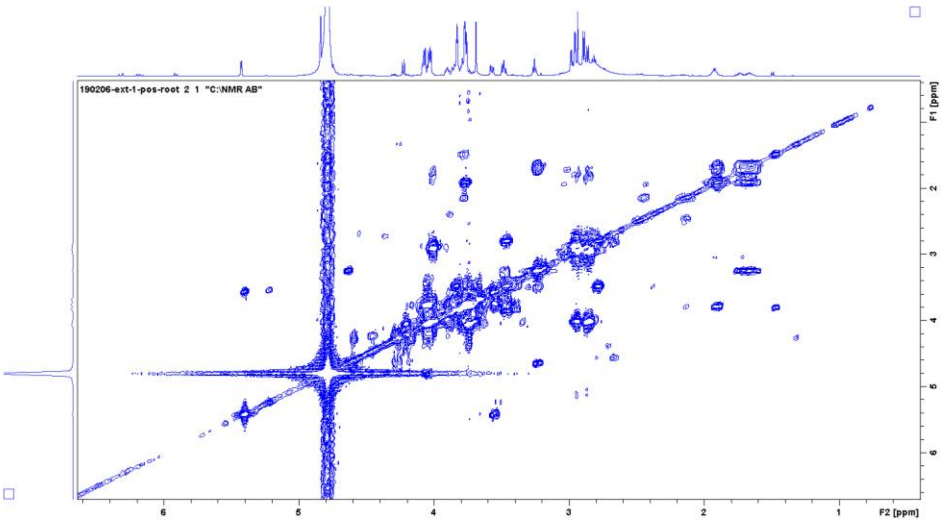

Figure S2 Extraction 1, 2D ^{1}H-^{1}H-NMR COSY of PO Roots

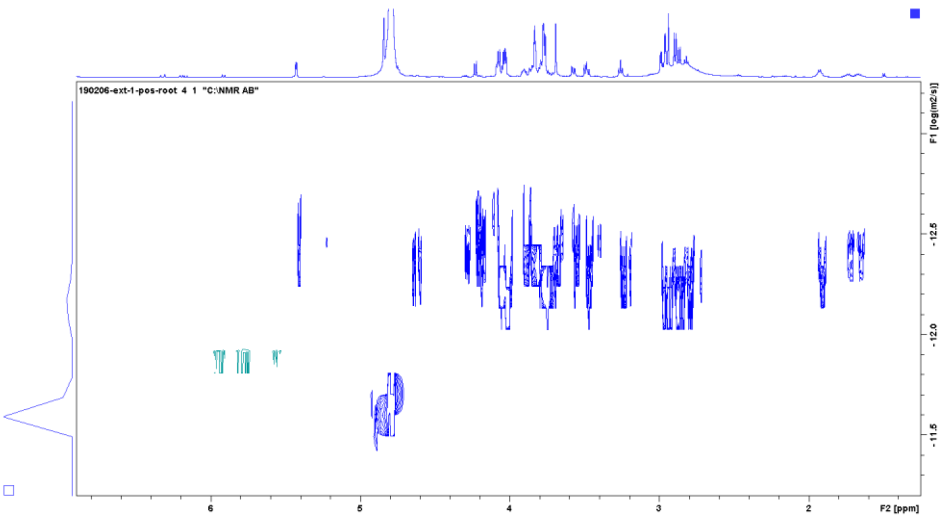

Figure S3 Extraction 1, 2D ^{1}H-^{1}H-NMR COSY of PO Roots

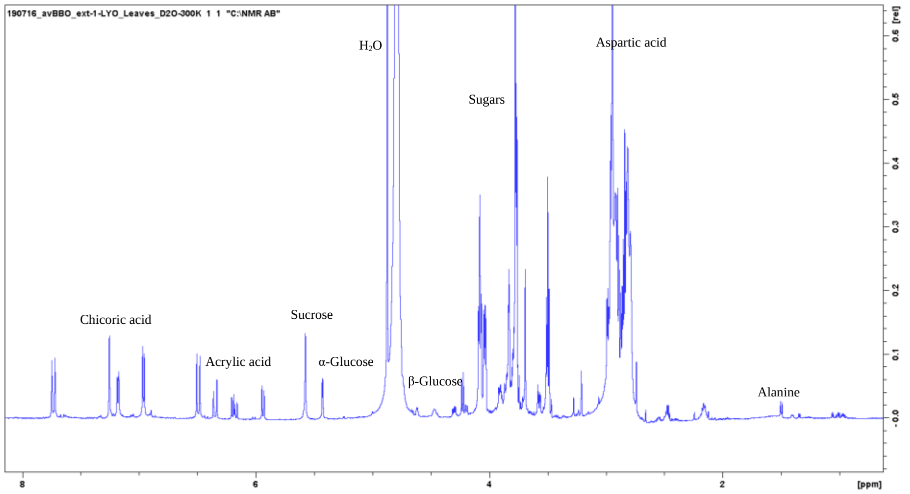

Figure S4 Extraction 1, 1D 1H-NMR of PO Leaves

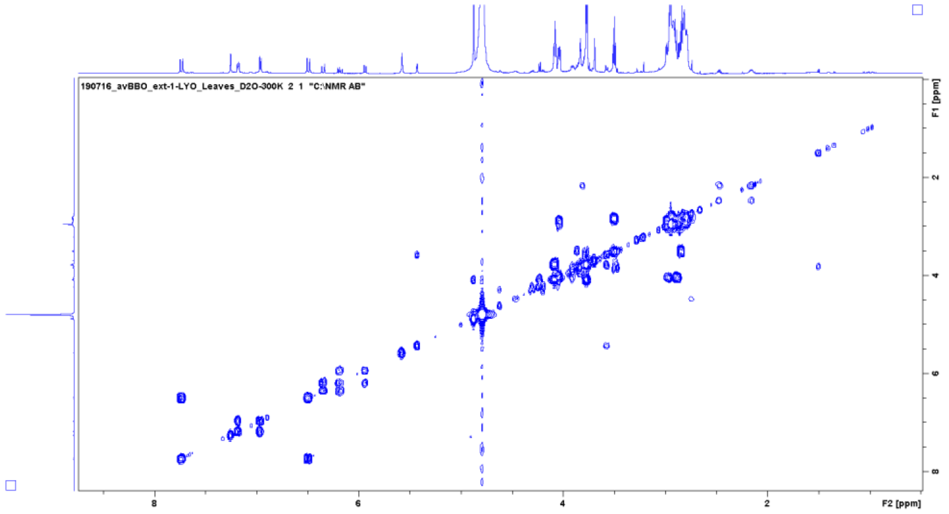

Figure S5 Extraction 1, 2D ^{1}H-^{1}H-NMR COSY of PO Leaves

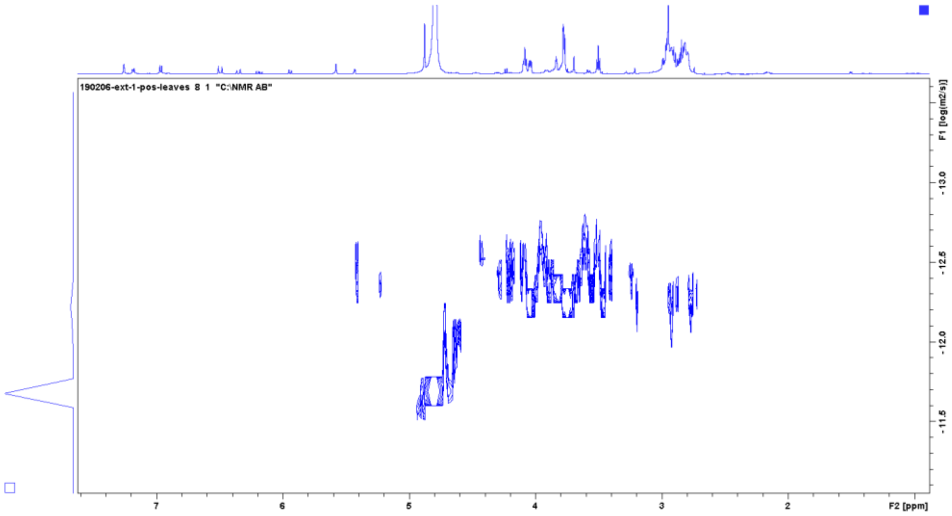

Figure S6 Extraction 1, 2D ^{1}H-^{1}H-NMR DOSY of PO Leaves

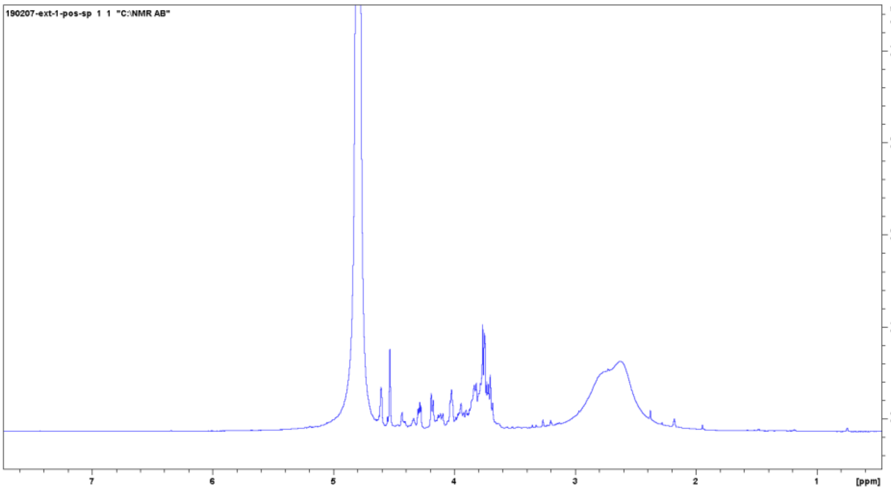

Figure S7 Extraction 1, 1D ^{1}H-NMR of PO Residues

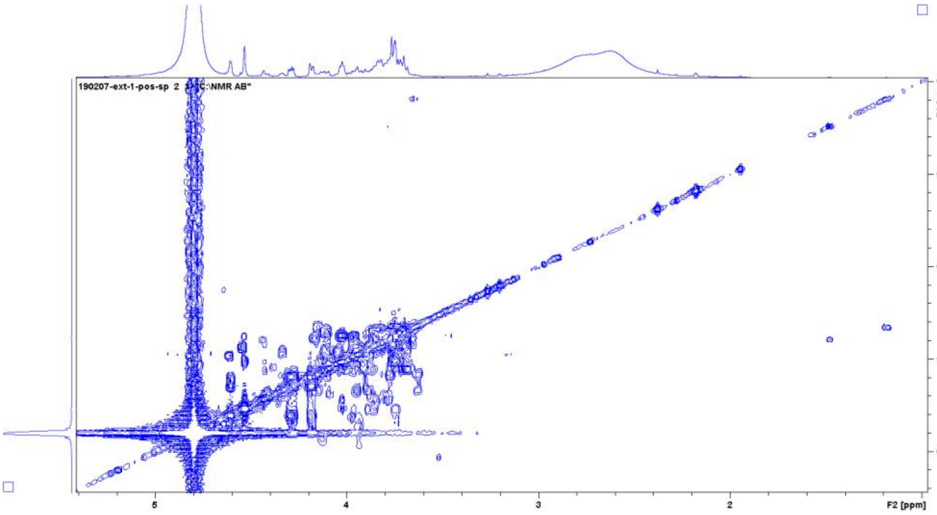

Figure S8 Extraction 1, 2D ^{1}H-^{1}H-NMR COSY of PO Residues

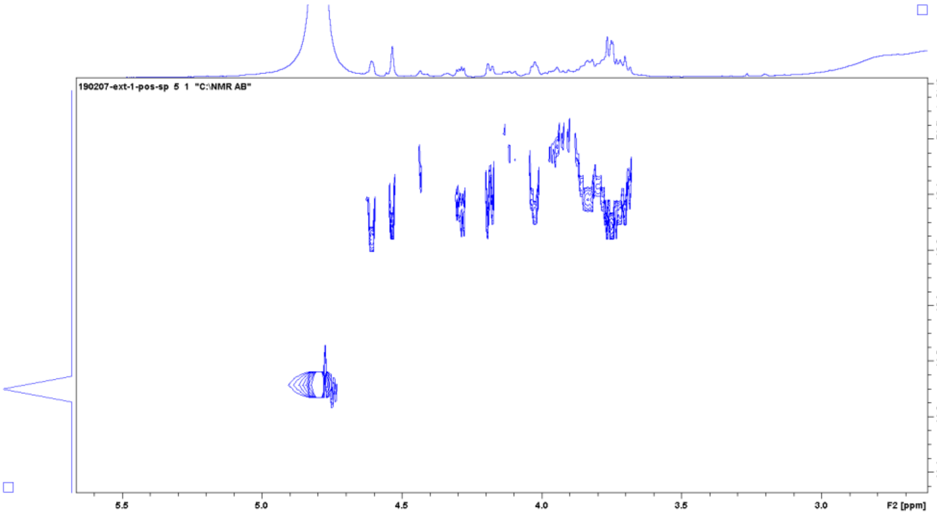

Figure S9 Extraction 1, 2D ^{1}H-^{1}H-NMR DOSY of PO Residues

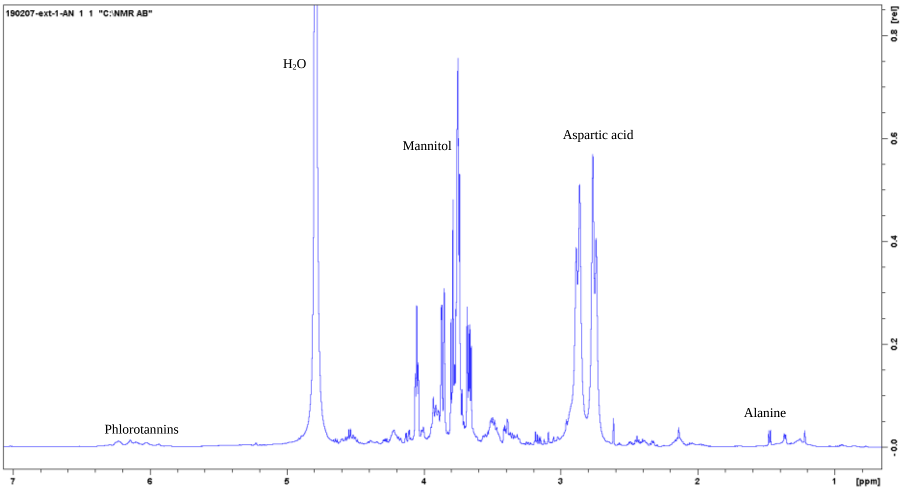

Figure S10 Extraction 1, 1D ^{1}H-NMR of AN

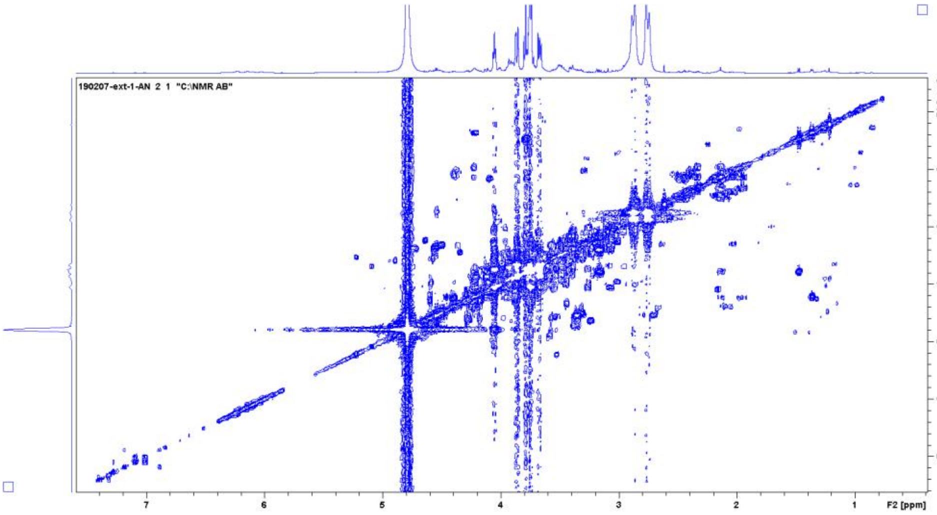

Figure S11 Extraction 1, 2D ^{1}H-^{1}H-NMR COSY of AN

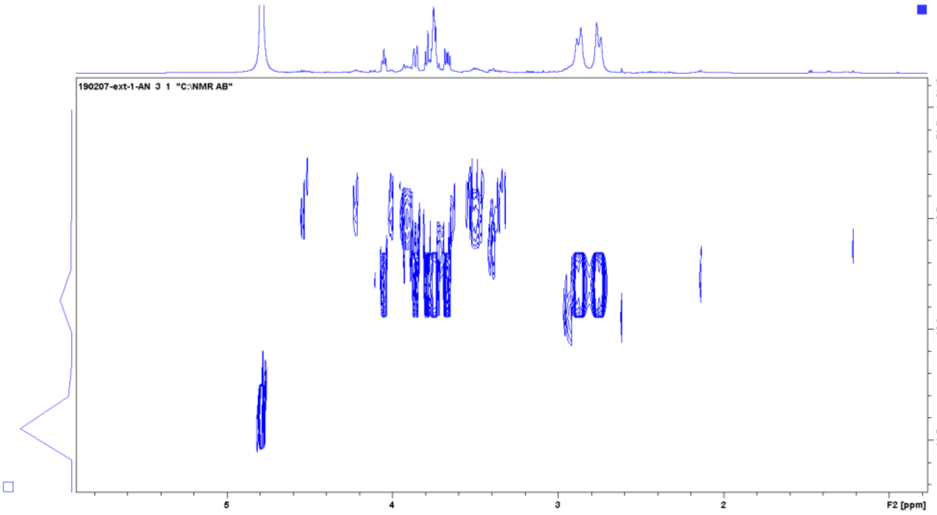

Figure S12 Extraction 1, 2D ^{1}H-^{1}H-NMR DOSY of AN

ESTRAZIONE 2: FIG.13-15 PO ROOTS, FIG. 16-18 PO LEAVES, FIG.19-21 PO RESIDUES, FIG.22-24 AN

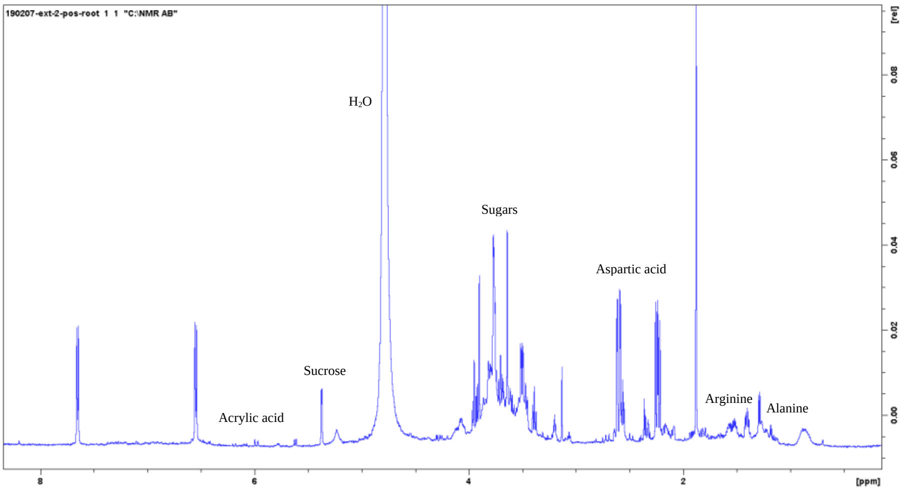

Figure S13 Extraction 2, 1D ^{1}H-NMR of PO Roots

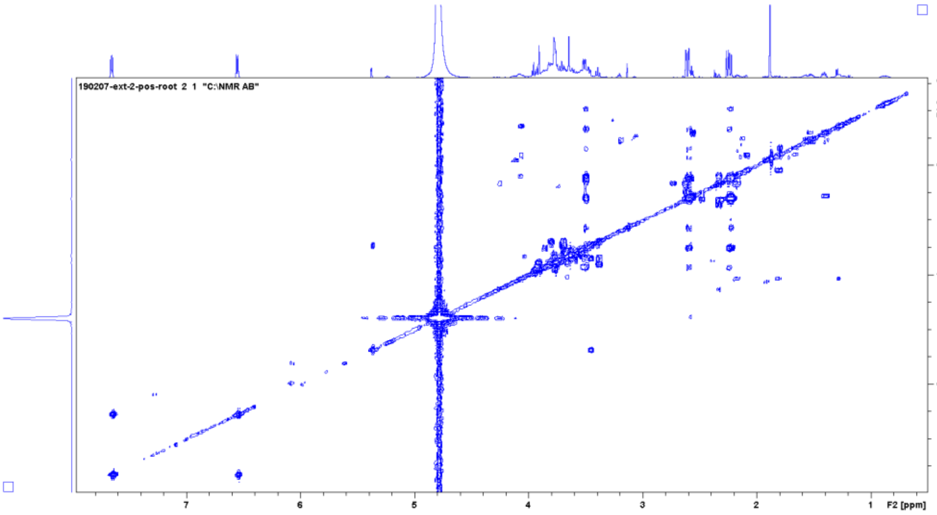

Figure S14 Extraction 2, 2D ^{1}H-^{1}H-NMR COSY of PO Roots

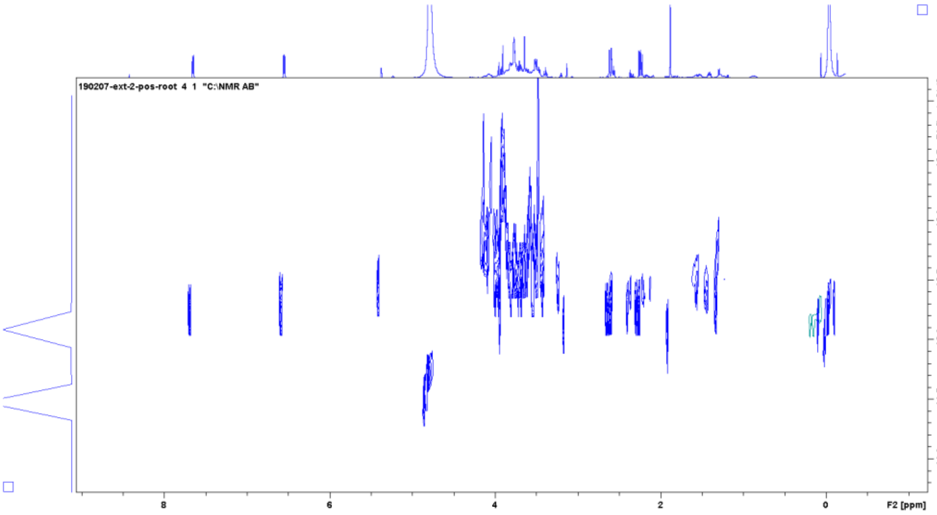

Figure S15 Extraction 2, 2D ^{1}H-^{1}H-NMR DOSY of PO Roots

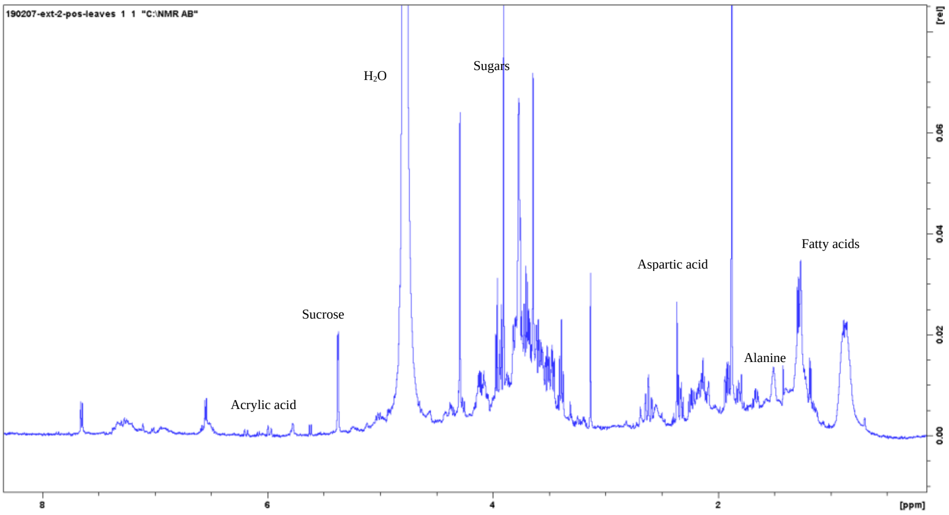

Figure S16 Extraction 2, 1D ^{1}H-NMR of PO Leaves

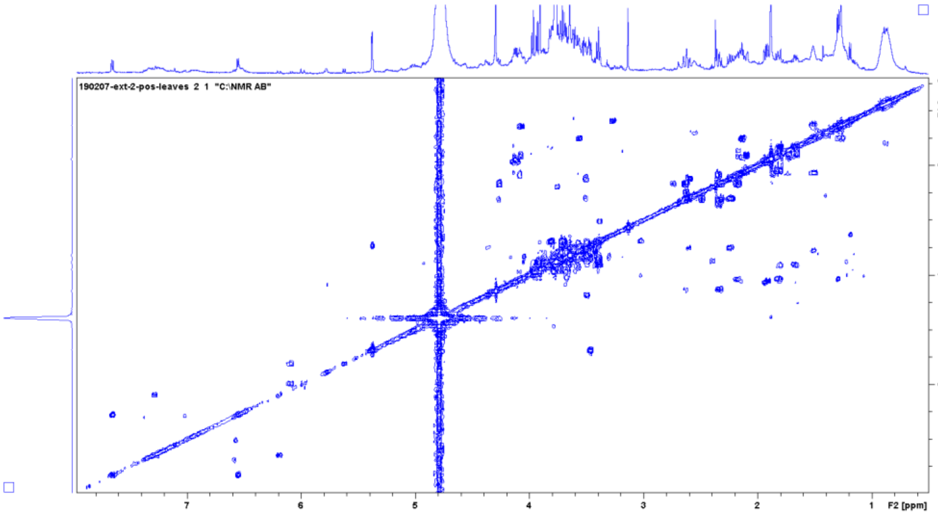

Figure S17 Extraction 2, 2D ^{1}H-^{1}H-NMR COSY of PO Leaves

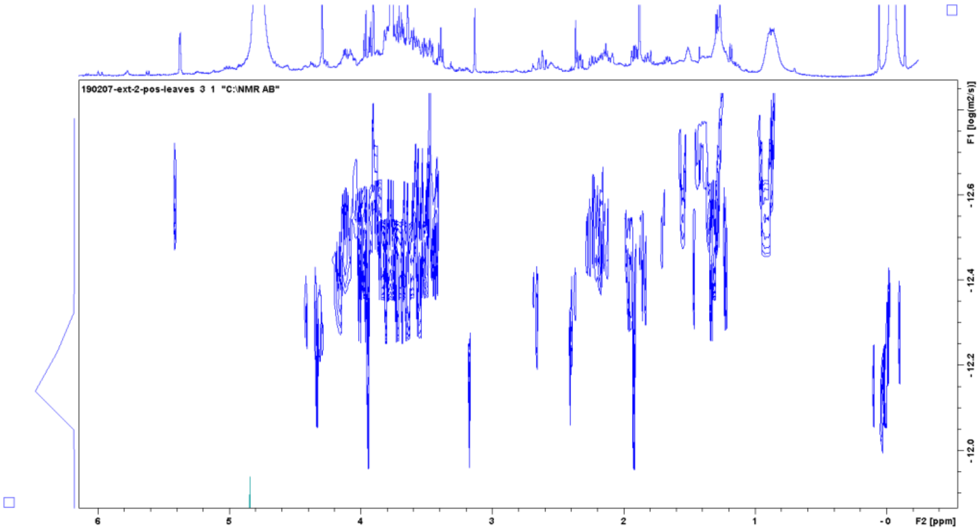

Figure S18 Extraction 2, 2D ^{1}H-^{1}H-NMR DOSY of PO Leaves

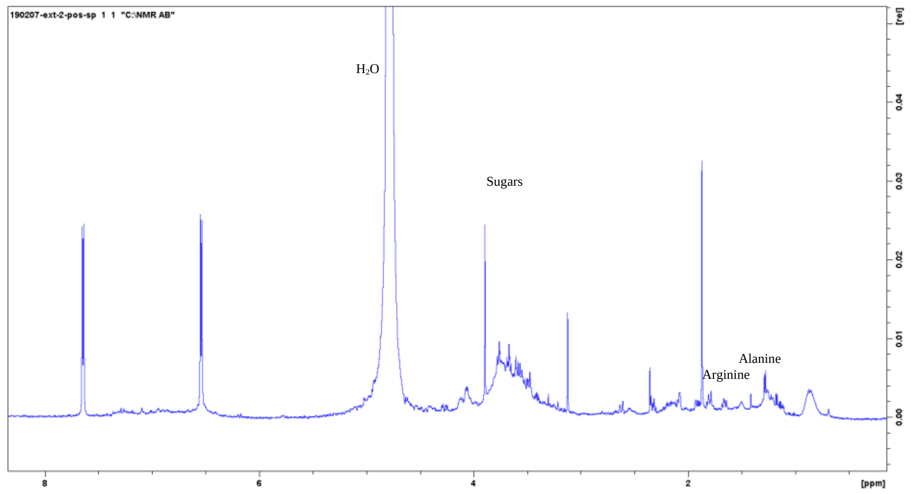

Figure S19 Extraction 2, 1D ^{1}H-NMR of PO Residues

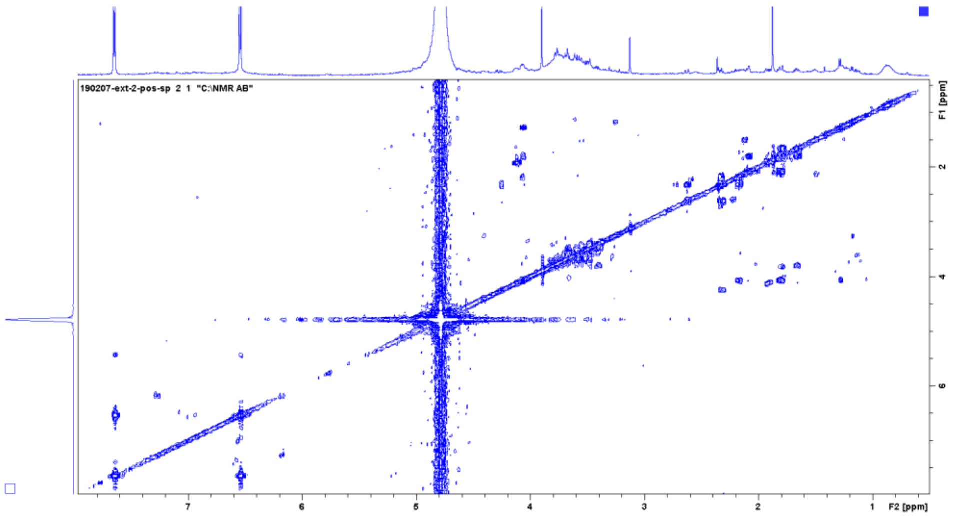

Figure S20 Extraction 2, 2D ^{1}H-^{1}H-NMR COSY of PO Residues

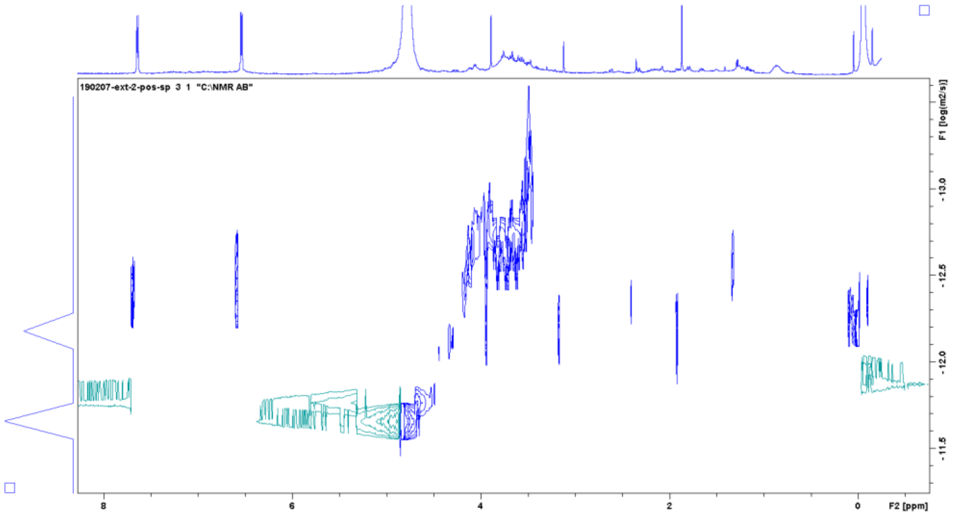

Figure S21 Extraction 2, 2D ^{1}H-^{1}H-NMR DOSY of PO Residues

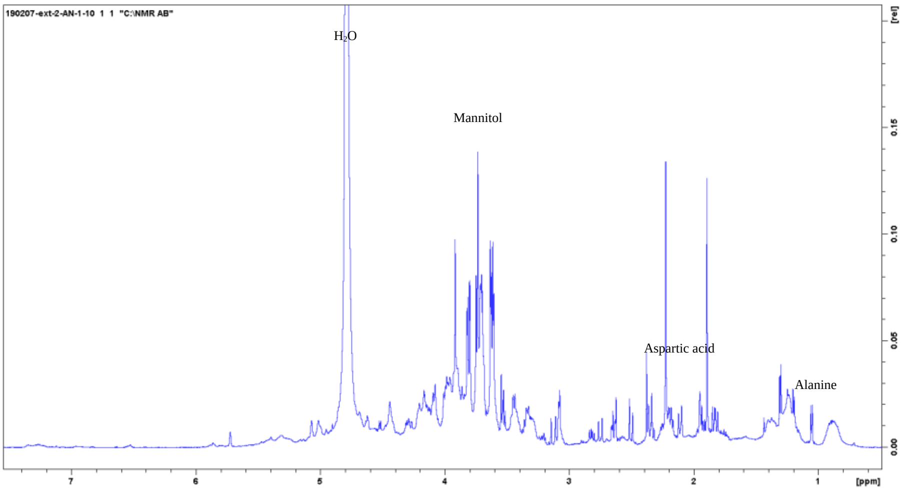

Figure S22 Extraction 2, 1D ^{1}H-NMR of AN

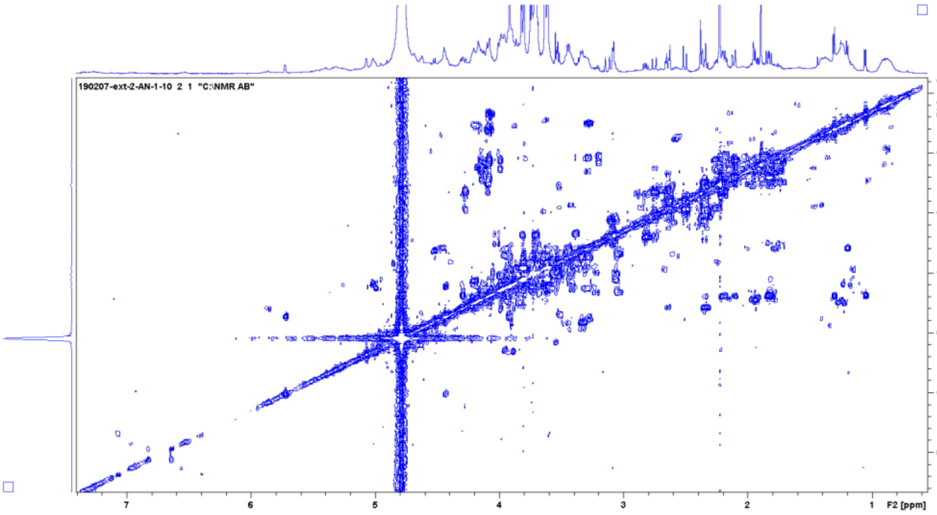

Figure S23 Extraction 2, 2D ^{1}H-^{1}H-NMR COSY of AN

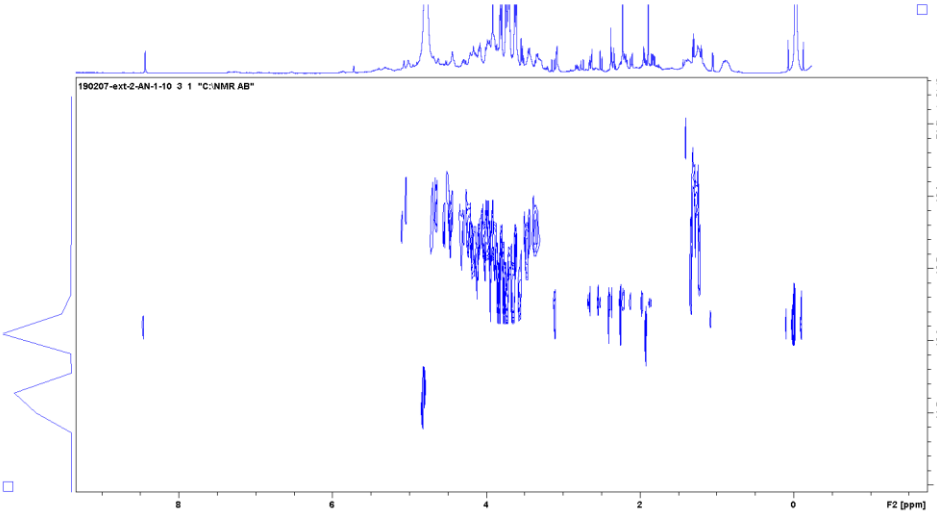

Figure S24 Extraction 2, 2D ^{1}H-^{1}H-NMR DOSY of AN

ESTRAZIONE 3: FIG.25-27 PO ROOTS, FIG. 28-30 PO LEAVES, FIG.31-33 PO RESIDUES, FIG.34-36 AN

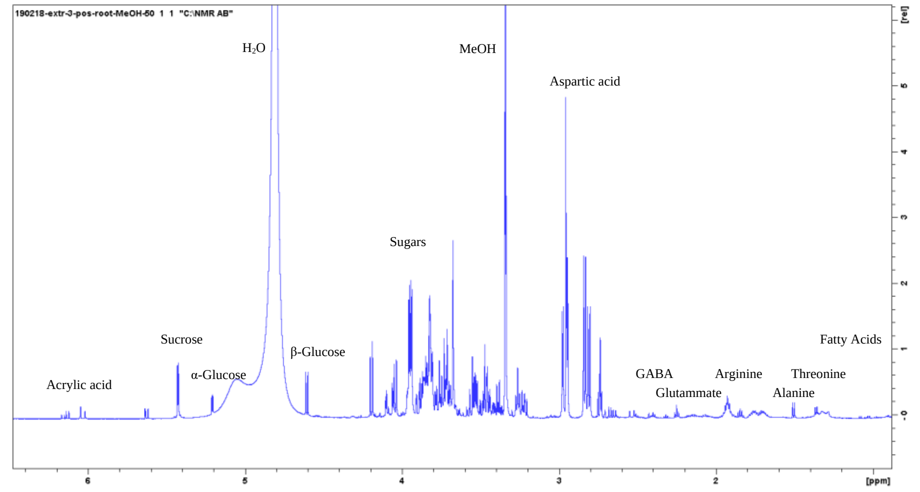

Figure S25 Extraction 3, 1D ^{1}H-NMR of Roots

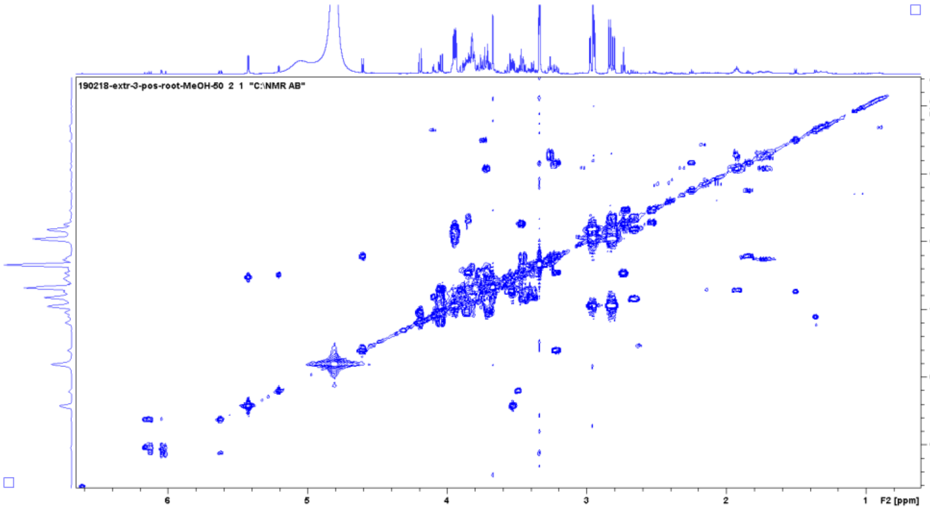

Figure S26 Extraction 3, 2D ^{1}H-^{1}H-NMR COSY of Roots

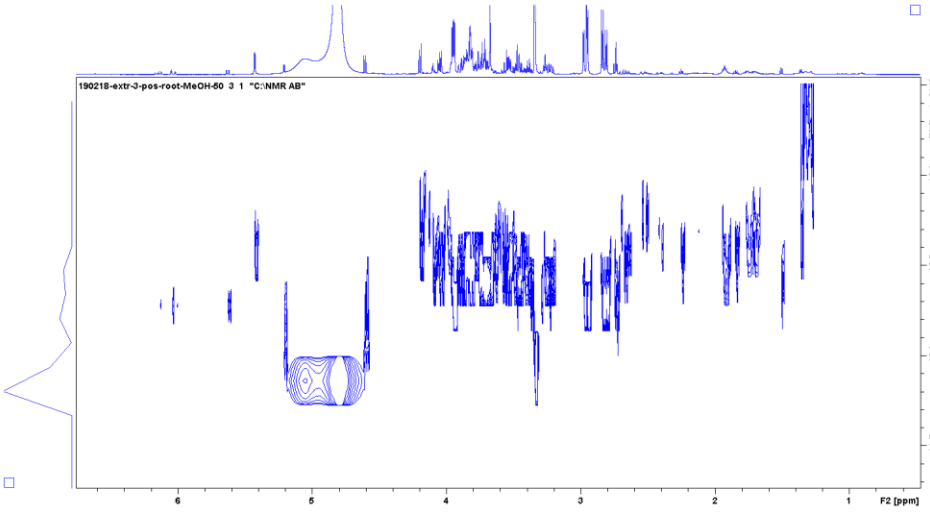

Figure S27 Extraction 3, 2D ^{1}H-^{1}H-NMR DOSY of PO Roots

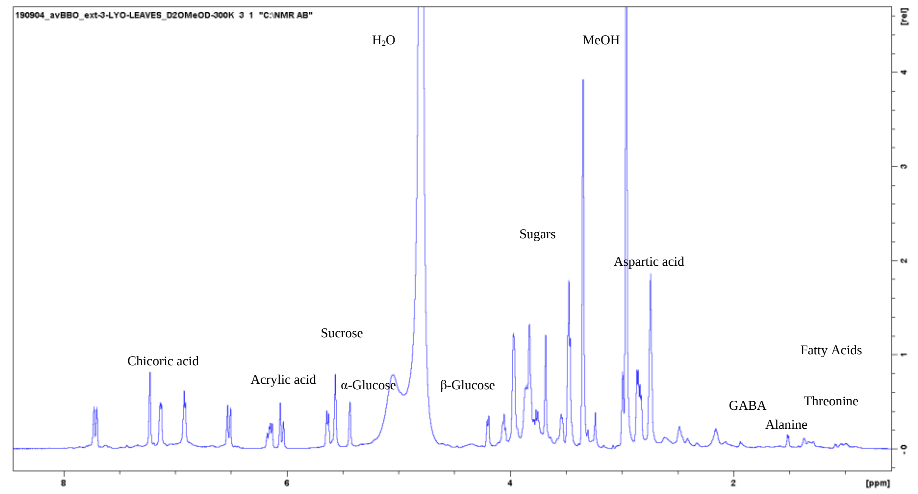

Figure S28 Extraction 3, 1D ^{1}H-NMR of PO Leaves

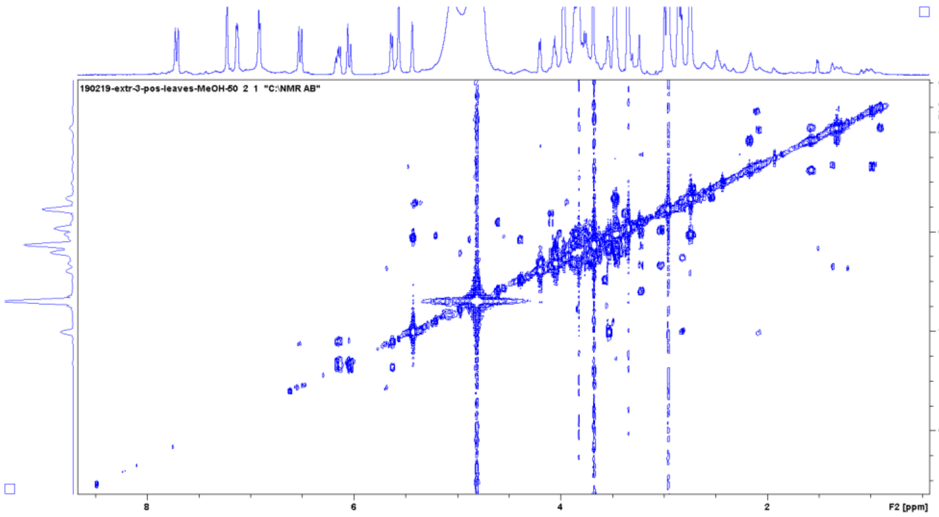

Figure S29 Extraction 3, 2D ^{1}H-^{1}H-NMR COSY of PO Leaves

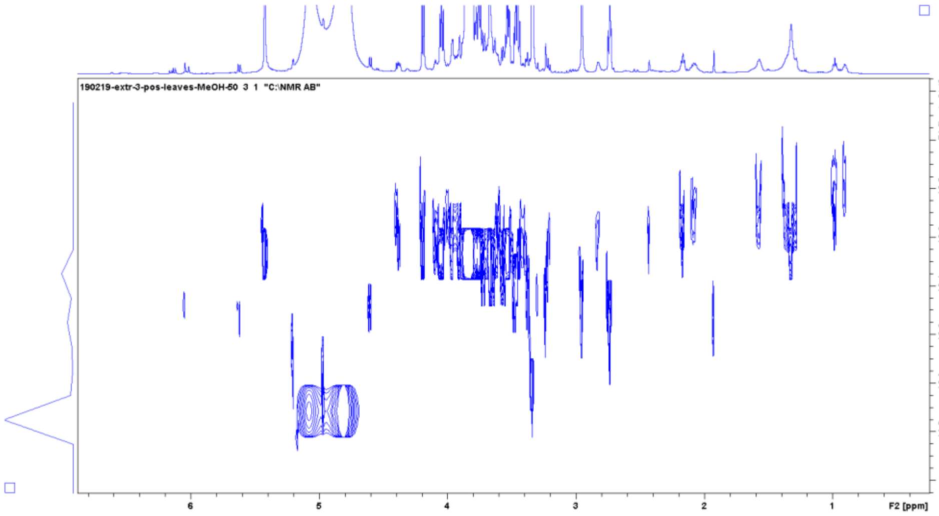

Figure S30 Extraction 3, 2D ^{1}H-^{1}H-NMR DOSY of PO Roots

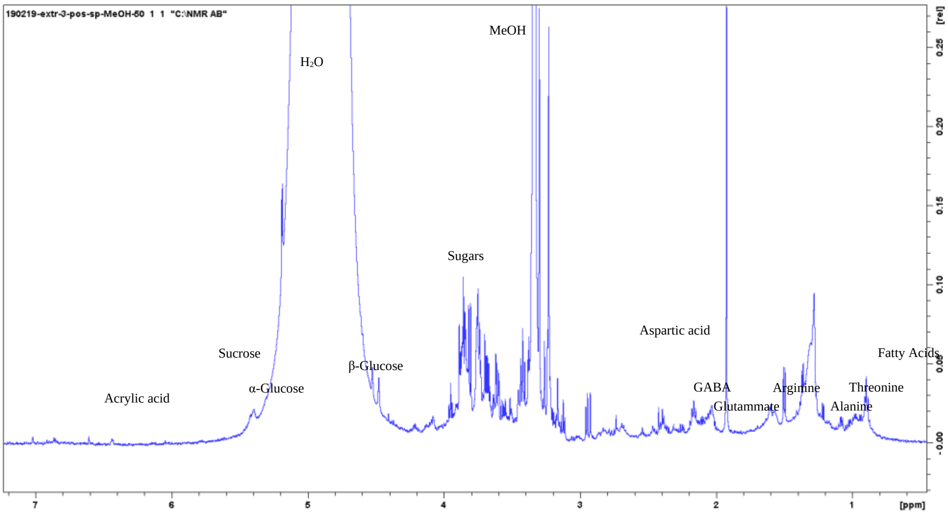

Figure S31 Extraction 3, 1D ^{1}H-NMR of PO Residues

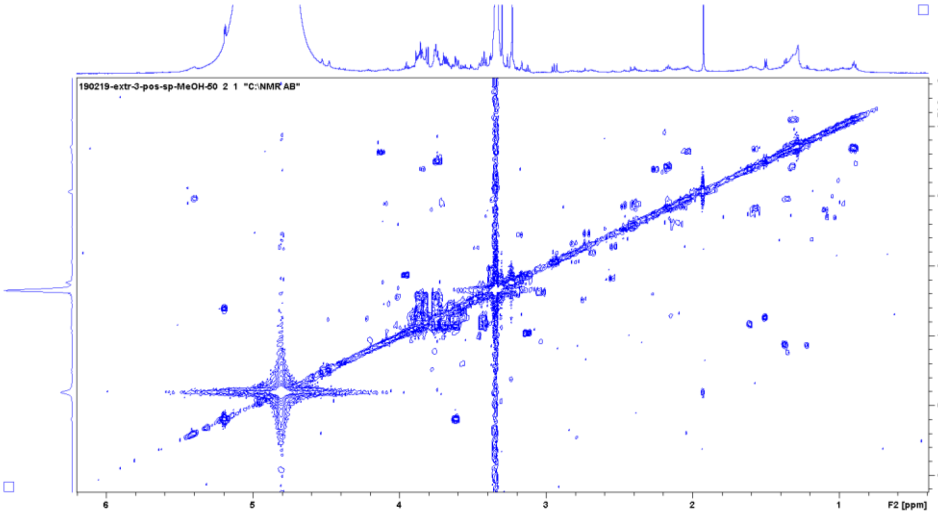

Figure S32 Extraction 3, 2D ^{1}H-^{1}H-NMR COSY of PO Residues

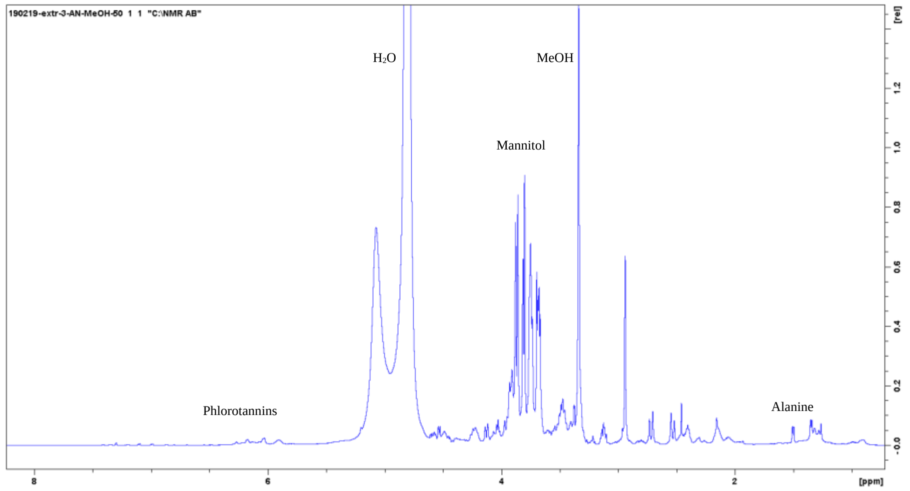

Figure S34 Extraction 3, 1D ^{1}H-NMR of AN

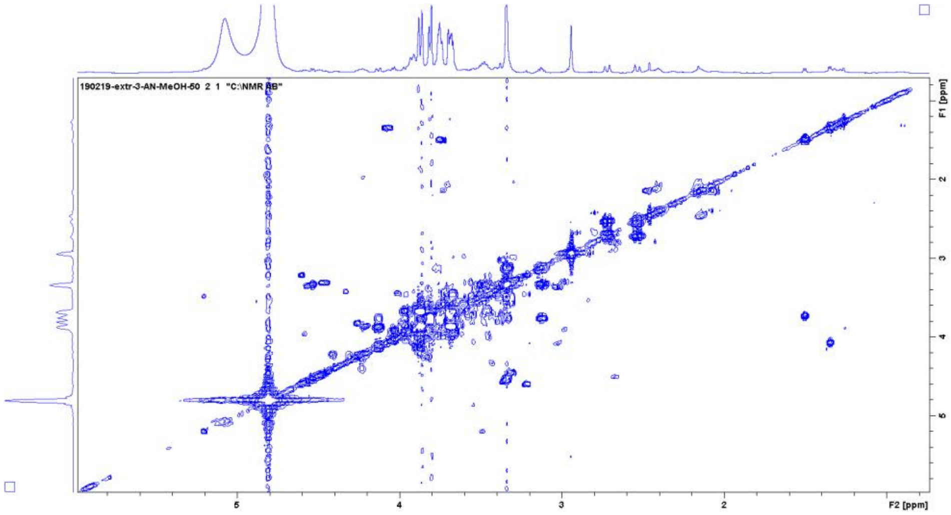

Figure S35 Extraction 3, 2D ^{1}H-^{1}H-NMR COSY of AN

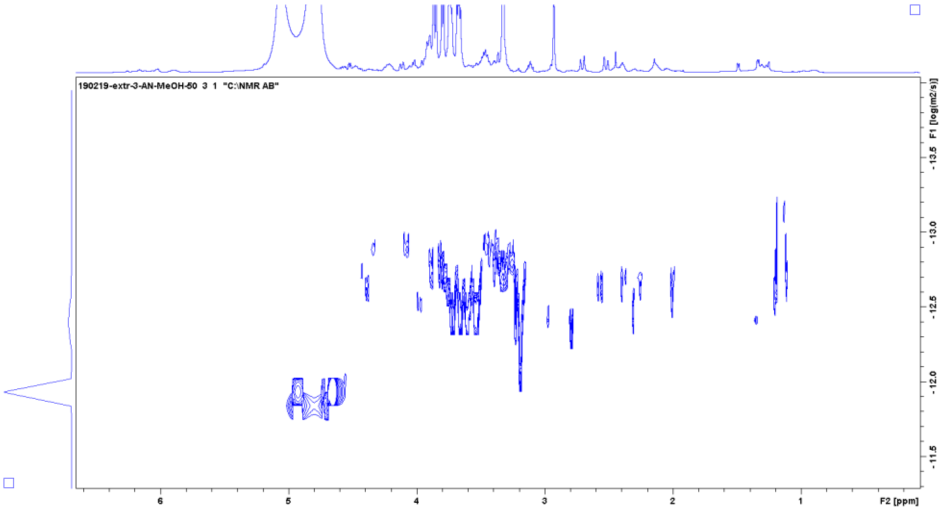

Figure S36 Extraction 3, 2D ^{1}H-^{1}H-NMR DOSY of AN

ESTRAZIONE 4: FIG.37-39 PO ROOTS, FIG. 40-42 PO LEAVES, FIG.43-45 PO RESIDUES, FIG.46-48 AN

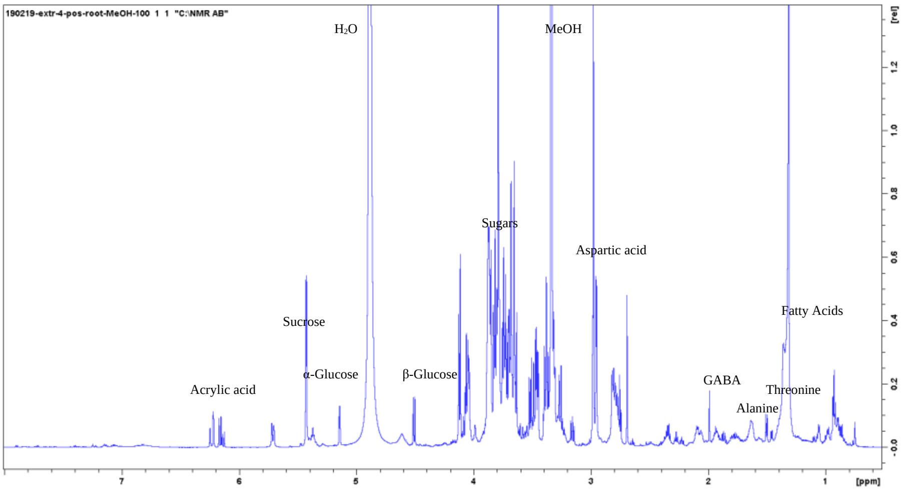

Figure S37 Extraction 4, 1D ^{1}H-NMR of PO Roots

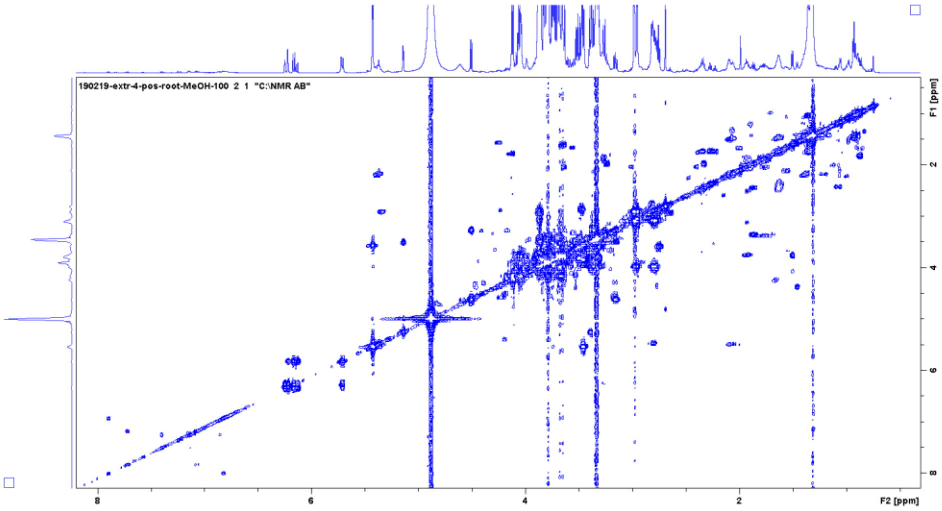

Figure S38 Extraction 4, 2D ^{1}H-^{1}H-NMR COSY PO Roots

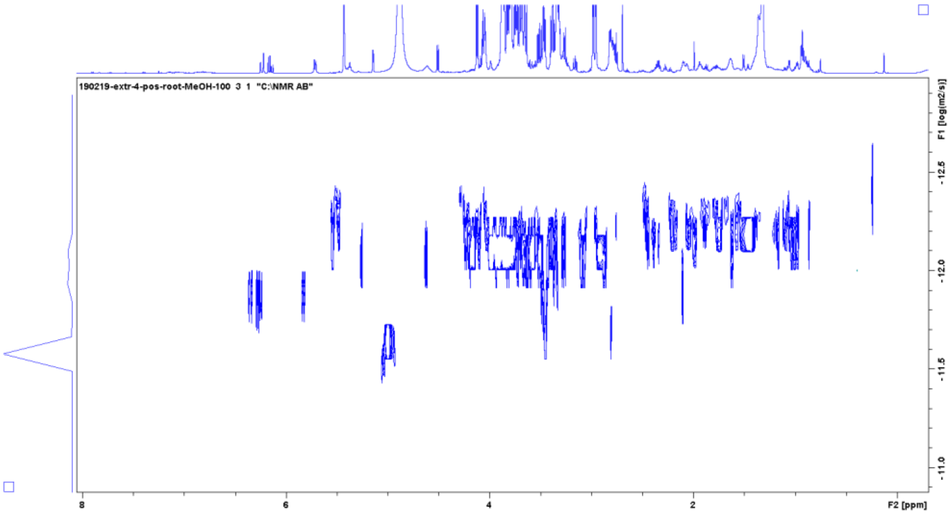

Figure S39 Extraction 4, 2D ^{1}H-^{1}H-NMR DOSY of PO Roots

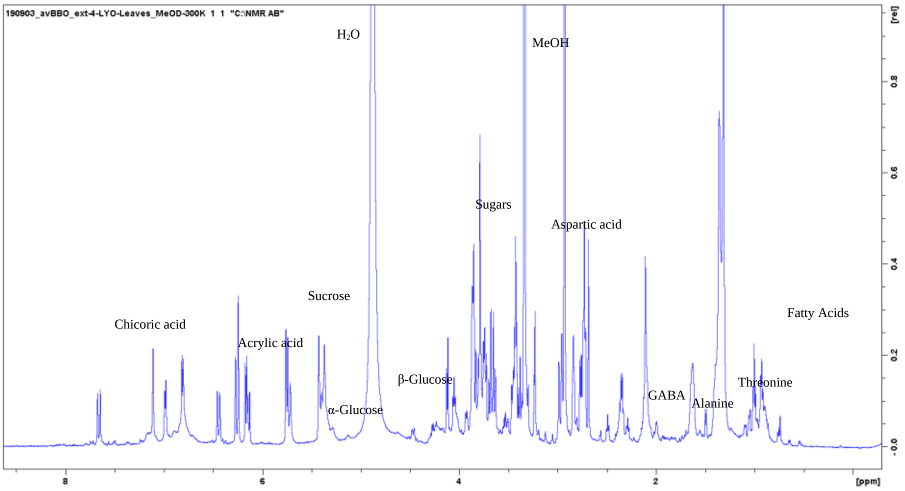

Figure S40 Extraction 4, 1D ^{1}H-NMR of PO Leaves

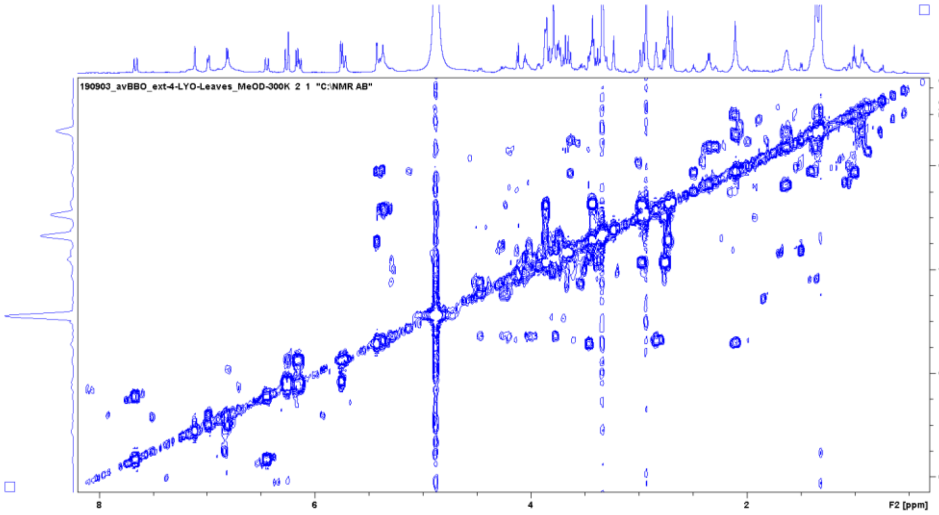

Figure S41 Extraction 4, 2D ^{1}H-^{1}H-NMR COSY PO Leaves

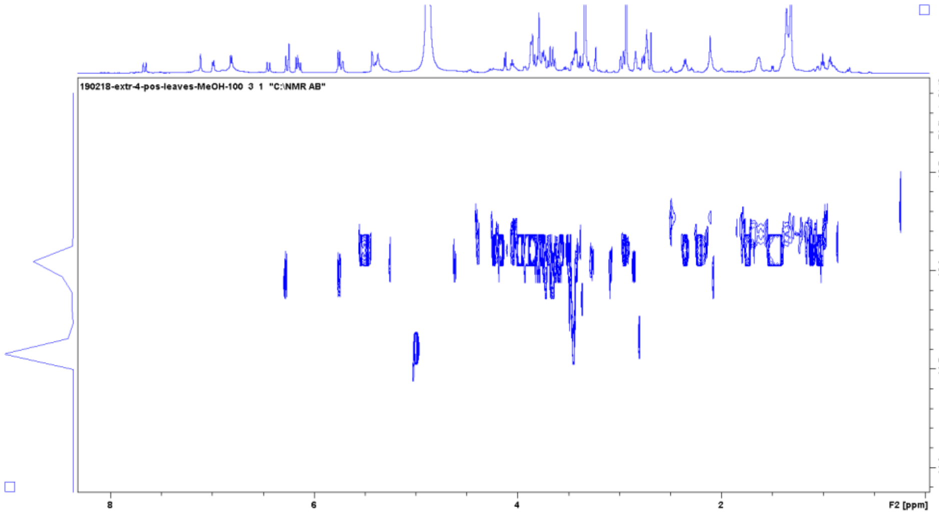

Figure S42 Extraction 4, 2D ^{1}H-^{1}H-NMR DOSY of PO Leaves

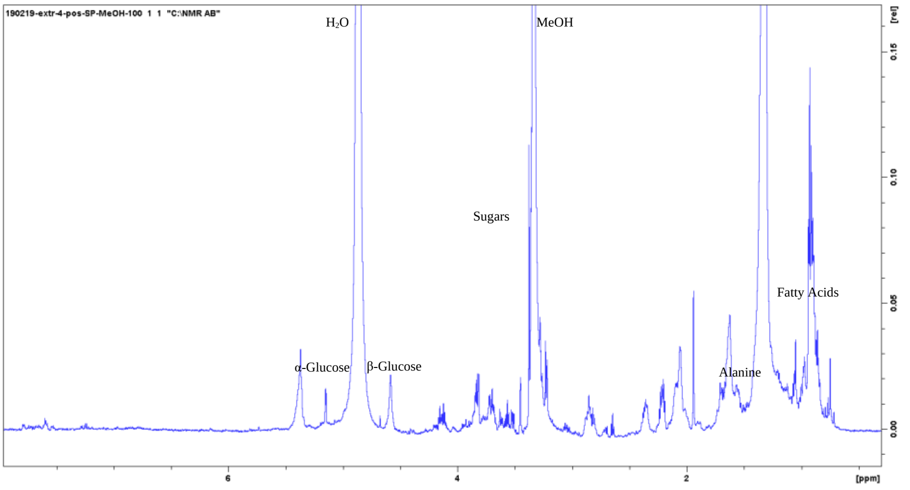

Figure S343 Extraction 4, 1D ^{1}H-NMR of PO Residues

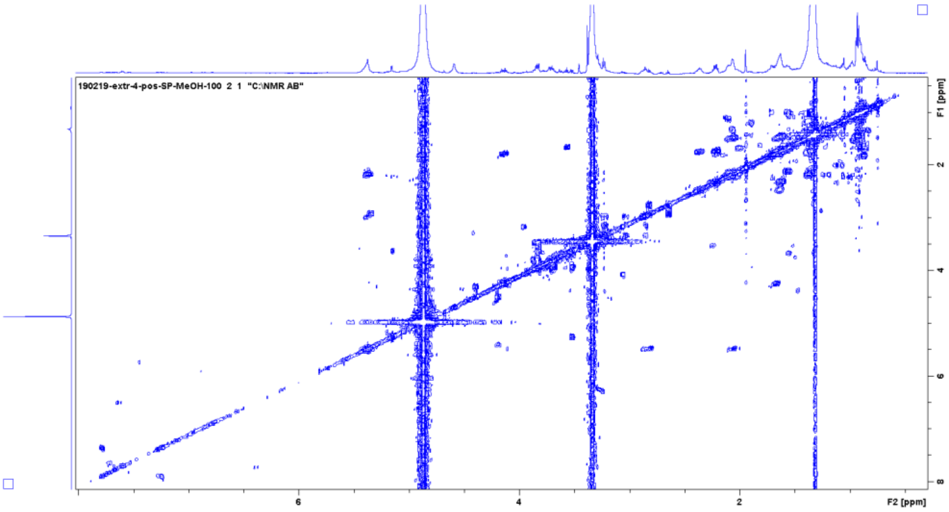

Figure S44 Extraction 4, 2D ^{1}H-^{1}H-NMR COSY PO Residues

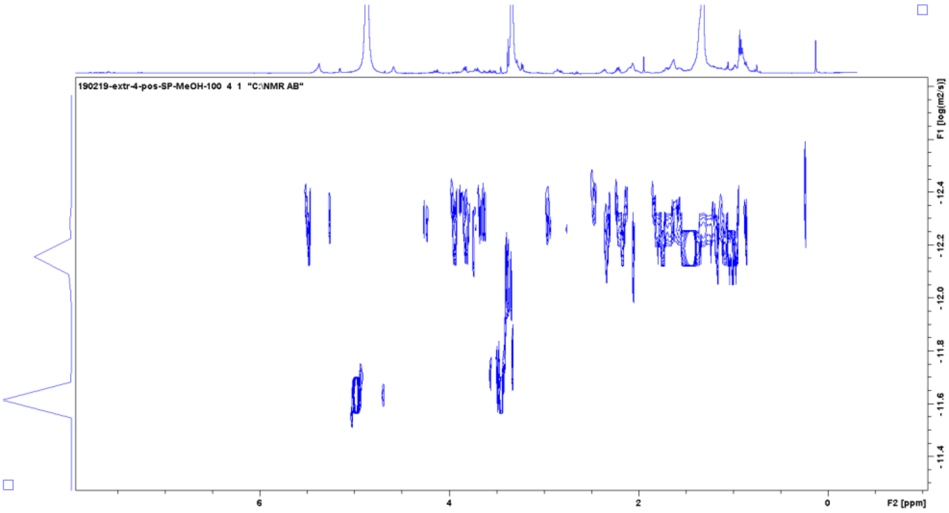

Figure S45 Extraction 4, 2D ^{1}H-^{1}H-NMR DOSY of PO Residues

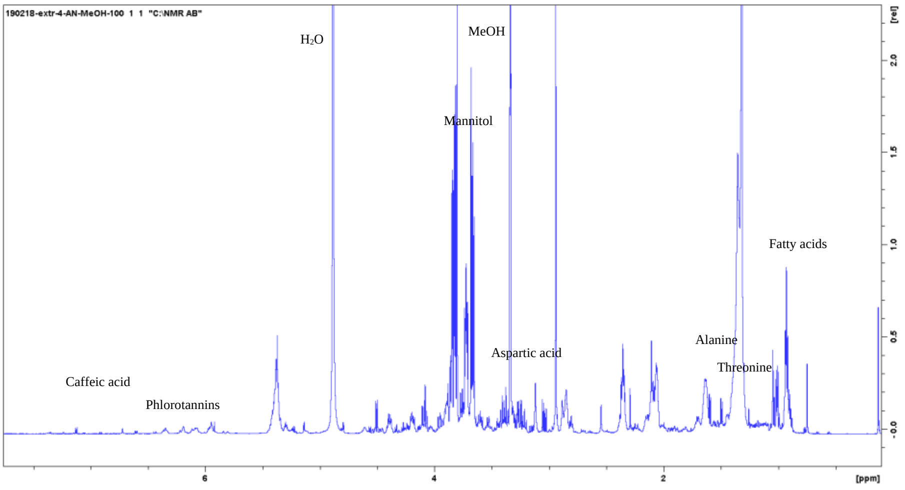

Figure S46 Extraction 4, 1D ^{1}H-NMR of AN

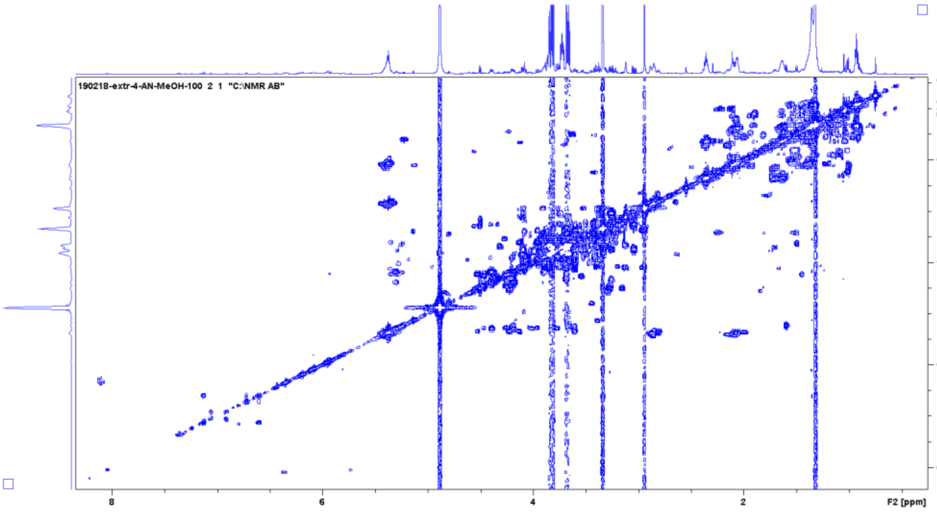

Figure S47 Extraction 4, 2D ^{1}H-^{1}H-NMR COSY AN

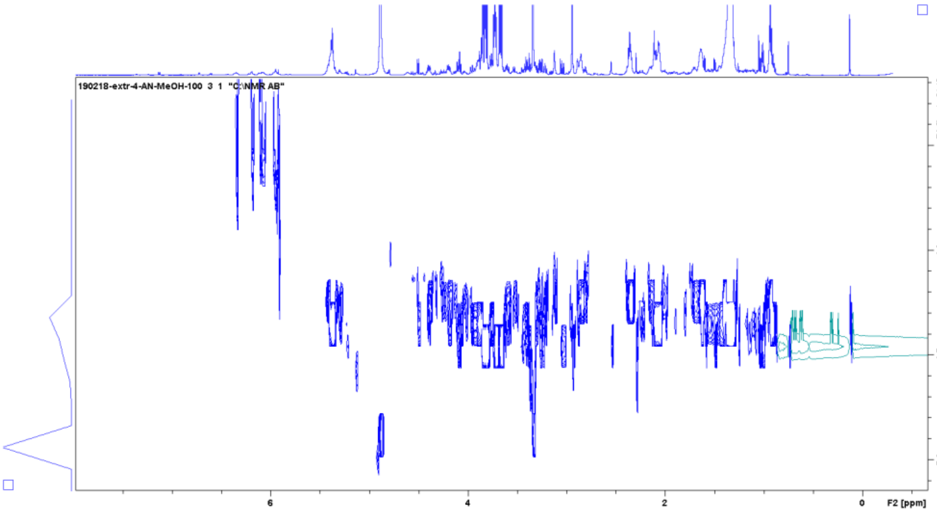

Figure S48 Extraction 4, 2D ^{1}H-^{1}H-NMR DOSY of AN
